# The binding property of a monoclonal antibody against the extracellular domains of aquaporin-4 directs aquaporin-4 toward endocytosis

**DOI:** 10.1016/j.bbrep.2016.05.017

**Published:** 2016-05-26

**Authors:** Ping Huang, Yoshiki Takai, Osamu Kusano-Arai, Julia Ramadhanti, Hiroko Iwanari, Takayuki Miyauchi, Toshiko Sakihama, Jing-Yan Han, Masashi Aoki, Takao Hamakubo, Kazuo Fujihara, Masato Yasui, Yoichiro Abe

**Affiliations:** a Department of Pharmacology, School of Medicine, Keio University, Tokyo, Japan; b Department of Neurology, Tohoku University School of Medicine, Sendai, Japan; c Quantitative Biology and Medicine, Research Center for Advanced Science and Technology, The University of Tokyo, Tokyo, Japan; d Keio Advanced Research Center for Water Biology and Medicine, Keio University, Tokyo, Japan; e Department Integration of Chinese and Western Medicine, Peking University Health Science Center, Beijing, China; f Tasly Microcirculation Research Center, Peking University Health Science Center, Beijing, China; g Key Laboratory of Microcirculation, State Administration of Traditional Chinese Medicine of the People’s Republic of China, Beijing, China; h Department of Multiple Sclerosis Therapeutics, Tohoku University Graduate School of Medicine, Sendai, Japan

**Keywords:** Aquaporin-4, Astrocyte, Autoimmune disease, Endocytosis, Monoclonal antibody, Neuromyelitis optica

## Abstract

Neuromyelitis optica (NMO), an autoimmune disease of the central nervous system, is characterized by an autoantibody called NMO-IgG that recognizes the extracellular domains (ECDs) of aquaporin-4 (AQP4). In this study, monoclonal antibodies (mAbs) against the ECDs of mouse AQP4 were established by a baculovirus display method. Two types of mAb were obtained: one (E5415A) recognized both M1 and M23 isoforms, and the other (E5415B) almost exclusively recognized the square-array-formable M23 isoform. While E5415A enhanced endocytosis of both M1 and M23, followed by degradation in cells expressing AQP4, including astrocytes, E5415B did so to a much lesser degree, as determined by live imaging using fluorescence-labeled antibodies and by Western blotting of lysate of cells treated with these mAbs. E5415A promoted cluster formation of AQP4 on the cell surface prior to endocytosis as determined by immunofluorescent microscopic observation of bound mAbs to astrocytes as well as by Blue native PAGE analysis of AQP4 in the cells treated with the mAbs. These observations clearly indicate that an anti-AQP4-ECDs antibody possessing an ability to form a large cluster of AQP4 by cross-linking two or more tetramers outside the AQP4 arrays enhances endocytosis and the subsequent lysosomal degradation of AQP4.

## Highlights

• Two mAbs against the ECD of mAQP4 with different binding properties was established.
• One of them, E5415A, bound to mAQP4 independent of OAP-formation of AQP4.
• E5415A but not E5415B strongly enhanced endocytosis of endogenous AQP4 in astrocytes.
• E5415A formed large clusters of AQP4 cross-linking multiple AQP4 functional units.
• It is the cluster formation of AQP4 that triggers AQP4 endocytosis.

## Graphical abstract

**Figure:**
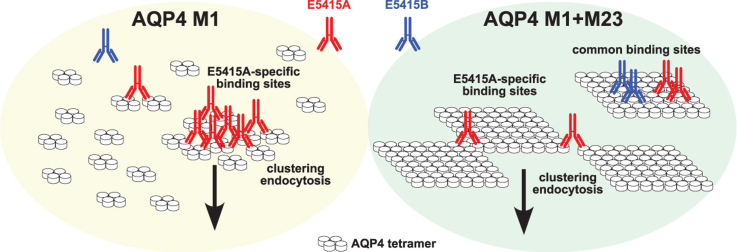

## 1 Introduction

Neuromyelitis optica (NMO) is an autoimmune disease of the central nervous system generally characterized by a disease-specific autoantibody called NMO-IgG [1]. A target of NMO-IgG is a water channel, aquaporin-4 (AQP4) [2], and binding of NMO-IgG to AQP4 expressed in astrocytic end-feet followed by complement-dependent and cell-mediated disruption of astrocytes causes demyelination in NMO [3], [4], [5], [6], [7]. NMO-IgG-induced endocytosis of AQP4 accompanied by excitatory amino acid transporter 2 (EAAT2), which leads to disruption of glutamate homeostasis and excitotoxicity of oligodendrocytes, was also proposed [7], [8].

AQP4 has two dominant isoforms—M1 and M23—both of which are simultaneously expressed and functions as a homo/heterotetramer randomly incorporating these two isoforms [32], [33]. AQP4 has a unique feature, namely formation of orthogonal arrays of particles (OAPs), in which AQP4 tetramers are orthogonally arranged [9], [10]. M23 lacks the initial 22 amino acids of the N-terminal cytoplasmic domain of M1 due to the difference in transcriptional start sites [11]. This 22-amino-acid domain interferes with formation of the OAPs [12]; therefore, M1 homotetramers form few arrays, while M23 homotetramers form much larger arrays than do endogenously expressed AQP4 homo/heterotetramers.

The epitopes for NMO-IgG in AQP4 are located in the extracellular domains (ECDs), consisting of three loops connecting the six transmembrane domains. In most cases, NMO-IgG preferentially binds to cells expressing M23 rather than to those expressing M1 alone, implying that OAP-formation of AQP4 contributes to recognition by NMO-IgG since the primary sequences of ECDs of M1 and M23 are identical [13], [14], [15], [16], [17], [18]. Multiple groups have reported that NMO-IgG only recognizes native AQP4 integrated into a lipid bilayer, suggesting that the epitope of NMO-IgG is formed by more than one loop in a conformational structure [13], [16], [23]. But some NMO-IgG can recognize denatured AQP4 as well as linear peptides corresponding to one of the extracellular loops [34].

NMO-IgG-induced endocytosis and/or degradation of AQP4 have been observed in some cell lines, including HEK293, CHO, and U87-MG, ectopically expressing AQP4. However, while a group demonstrated that NMO-IgG only induced endocytosis of M1, the other group reported that recombinant NMO-IgGs cloned from cerebrospinal fluid plasmablasts of an NMO patient induced endocytosis of both isoform [8], [19], [20], [21]. In addition to AQP4 over-expressed in a stable cell line, NMO-IgG–induced endocytosis of endogenous AQP4 in a primary culture of rat, mouse, and human astrocytes was also reported [8], [20], [22]. On the other hand, one of the recombinant monoclonal NMO-IgGs, rAb-53 induced little endocytosis of endogenous mouse AQP4 (mAQP4) in primary cultured astrocytes [15], [21], [23], despite the fact that it did endocytosis of ectopically expressed AQP4 in a stable cell line [21]. These conflicting findings may be attributable to differences in the binding properties of each anti-AQP4 antibody contained in the sera of NMO patients because as mentioned above, epitopes of NMO-IgG vary among patients. One difficulty in analyzing the binding properties of NMO-IgGs in patient sera is that they are ‘polyclonal’ ones containing multiple NMO-IgGs with a variety of binding characteristics as well as other unrelated antibodies. Therefore, it is impossible to address this question quantitatively unless each anti-AQP4 antibody is cloned from patient plasma cells, as Bennett et al. did previously [23].

In this study, we developed two NMO-IgG-like monoclonal antibodies (mAbs) against the ECDs of mAQP4 using a baculovirus display method [24]: one recognizing both M1 and M23, and the other almost exclusively recognizing M23. Using these antibodies, we examined the relationship between their binding properties and subsequent endocytosis of AQP4 in astrocytes.

## 2 Materials and methods

### 2.1 Development of mAbs against the ECDs of mAQP4

mAbs against the ECDs of mAQP4 were developed using a baculovirus display method [24], [25]. To circumvent the immunological tolerance for mAQP4, gp64 transgenic mice [24] with an AQP4 knockout genetic background [26] (Acc. No. CDB0758K, http://www.cdb.riken.jp/arg/mutant%20mice%20list.html) were used as hosts. After screening hybridoma culture supernatants by flow cytometry and an ELISA using CHO cells stably expressing mAQP4 M23 (Supplemental Fig. S1A, lane 1), eight clones were obtained (Supplemental Fig. S1B).

### 2.2 Mice and primary culture of astrocytes

Primary cultured astrocytes derived from C57BL/6 J mice were prepared as described previously [27]. Animal experiments were performed according to the Guidelines for the Care and Use of Laboratory Animals of Keio University School of Medicine (09084‐7).

### 2.3 Western blotting

Western blotting was performed as described [28]. To confirm lysosomal degradation of AQP4 in astrocytes, 500 nM bafilomycin A1 (Merck Millipore, Billerica, MA, USA) [29], was added to culture media and incubated for 24 h. Antibodies used were monoclonal anti-AQP4 (E5206, 1:2000, ref 25); rabbit polyclonal anti-AQP4 (Sigma, St Louis, MO); and monoclonal anti-actin (2F3, 1:5000, Wako Pure Chemical Industries).

### 2.4 Blue native PAGE

To determine the size of complexes containing AQP4 in cells treated with mAbs, Blue native PAGE (BN-PAGE) was performed as previously reported [30]. Stable CHO-cell lines and mouse astrocytes were incubated with either E5415A or E5415B (1.5 μg/ml) for 3 h. Afterwards, cells were lysed in NativePAGE sample buffer (Life Technologies, Carlsbad, CA, USA) containing 1% n-dodecyl-β-d-maltoside (DDM). The lysates were centrifuged to collect the supernatants (DDM-soluble fraction), which was mixed with 5% Coomassie Brilliant Blue G-250 (detergent: G-250 ratio of 4:1), loaded in polyacrylamide native gradient gels (3–12%), and electrophoresed. Protein precipitated with the pellets (DDM-insoluble fraction) was extracted with SDS sample buffer and subjected to Western blotting. AQP4 and mouse IgG were detected with rabbit antibody against the C-terminal domain followed by HRP-conjugated anti-rabbit IgG and HRP-conjugated anti-mouse IgG, respectively.

### 2.5 Confocal microscopy

E5415A and E5415B were labeled with Alexa Fluor 555 as described [29]. To examine endocytosis of AQP4 in astrocytes, primary cultured astrocytes were plated onto 3.5-cm glass base dishes and grown for 3–4 days. For endosomal localization of mAbs, cells were preincubated with 100 µg/ml Alexa-Fluor-488-conjugated transferrin (Life Technologies) in Hanks' BSS for 10 min. Cells were then incubated with 2 µg/ml Alexa-Fluor-555-labeled E5415A or E5415B for up to 3 h. For lysosomal localization, astrocytes were incubated with either Alexa-Fluor-555-labeled mAb in the presence of the lysosomal marker LysoTracker Green DND-26 (1 µM, Life Technologies) for 3 or 24 h.

To distinguish between mAbs bound to cell-surface and to intracellular AQP4, primary cultured astrocytes were incubated with 2 µg/ml of each mAb at 37 °C for the indicated times. Cells were then cooled to 4 °C to avoid further endocytosis and washed with ice-cold PBS. mAb bound to surface AQP4 was labeled with Alexa-Fluor-488-conjugated goat anti-mouse IgG (Life Technologies) at 4 °C for 1 h. Subsequently, cells were fixed with 4% PFA, washed with PBS, and permeabilized with 0.3% Triton X-100 in PBS. mAb bound to intracellular AQP4 was then labeled with Alexa-Fluor-555-conjugated goat anti-mouse IgG (Life Technologies) at 4 °C for 1 h.

### 2.6 Statistical analysis

Statistical analysis was performed using JMP ver. 11.0.0 (SAS Institute Inc., Cary, NC, USA). Data were analyzed using one-way ANOVA followed by the Tukey-Kramer method.

## 3 Results and discussion

### 3.1 Development of mAbs recognizing the ECDs of mAQP4

We developed mAbs against the ECDs of mAQP4 (Supplemental Fig. S1B), which mimic NMO-IgG. Similarly to NMO-IgGs we have examined so far [13] as well as those other groups have demonstrated [16], [23], these mAbs did not recognize denatured AQP4 as determined by Western blotting of lysate derived from mouse cerebellum (data not shown). Among these clones, we chose E5415A and E5415B as clones recognizing both M1 and M23 isoforms and almost exclusively recognizing M23, respectively, for the subsequent experiments.

### 3.2 Effects of mAbs against the ECD of AQP4 on the endocytosis of AQP4 in astrocytes

**Fig. 1:**
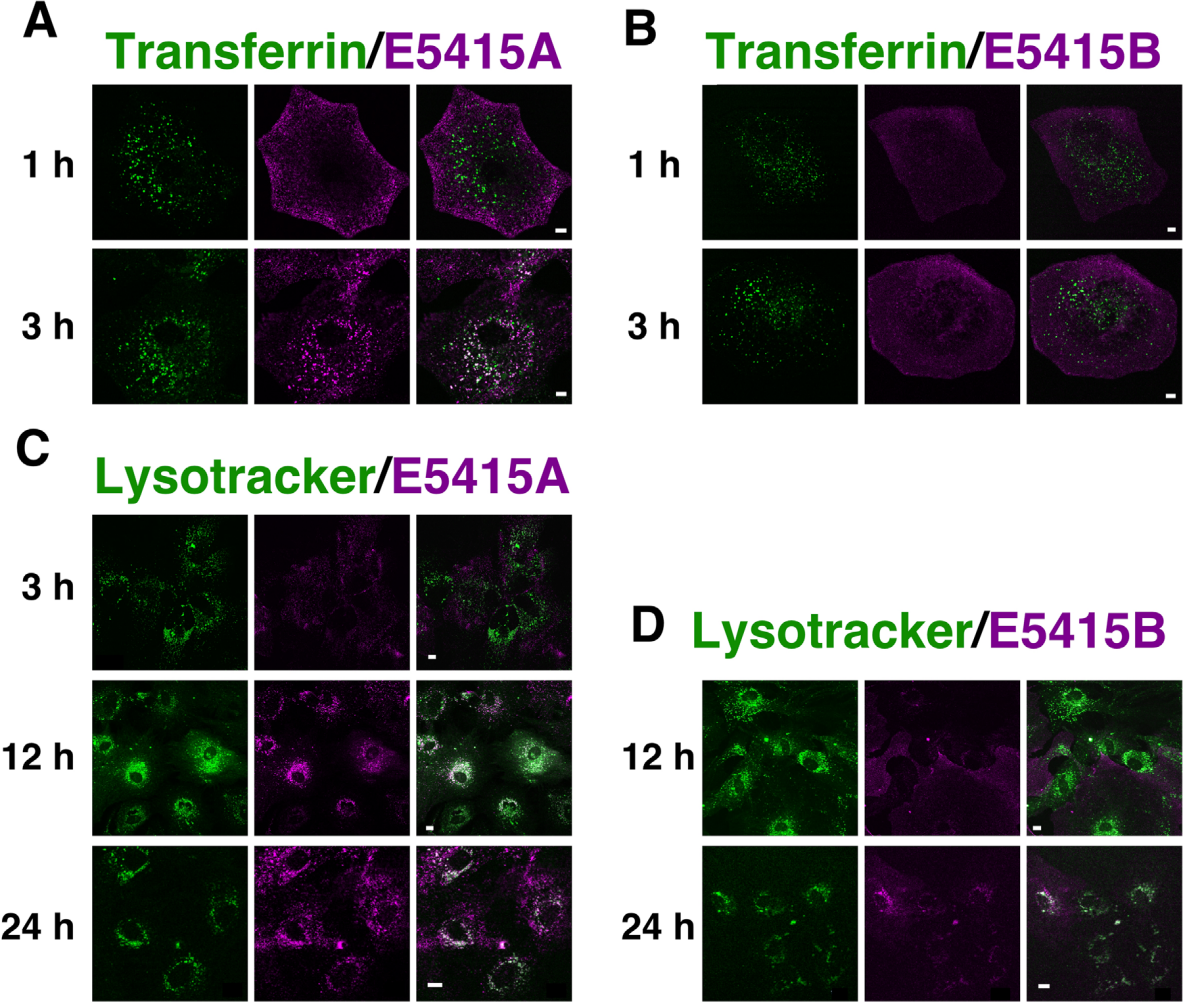
Induction of endocytosis of AQP4 by mAbs in primary cultured astrocytes. (A and B) Localization of fluorescence-labeled E5415A (A, magenta) or E5415B (B, magenta) and Alexa-Fluor-488-labeled transferrin (green) in primary cultured astrocytes was examined by confocal microscopy up to 3 h. Bar=5 µm. (C and D) Localization of fluorescence-labeled E5415A (C, magenta) or E5415B (D, magenta) and Lysotracker 488 (green) in primary cultured astrocytes was examined by confocal microscopy after incubation for 12 and 24 h. Bar=10 µm.

**Fig. 2:**
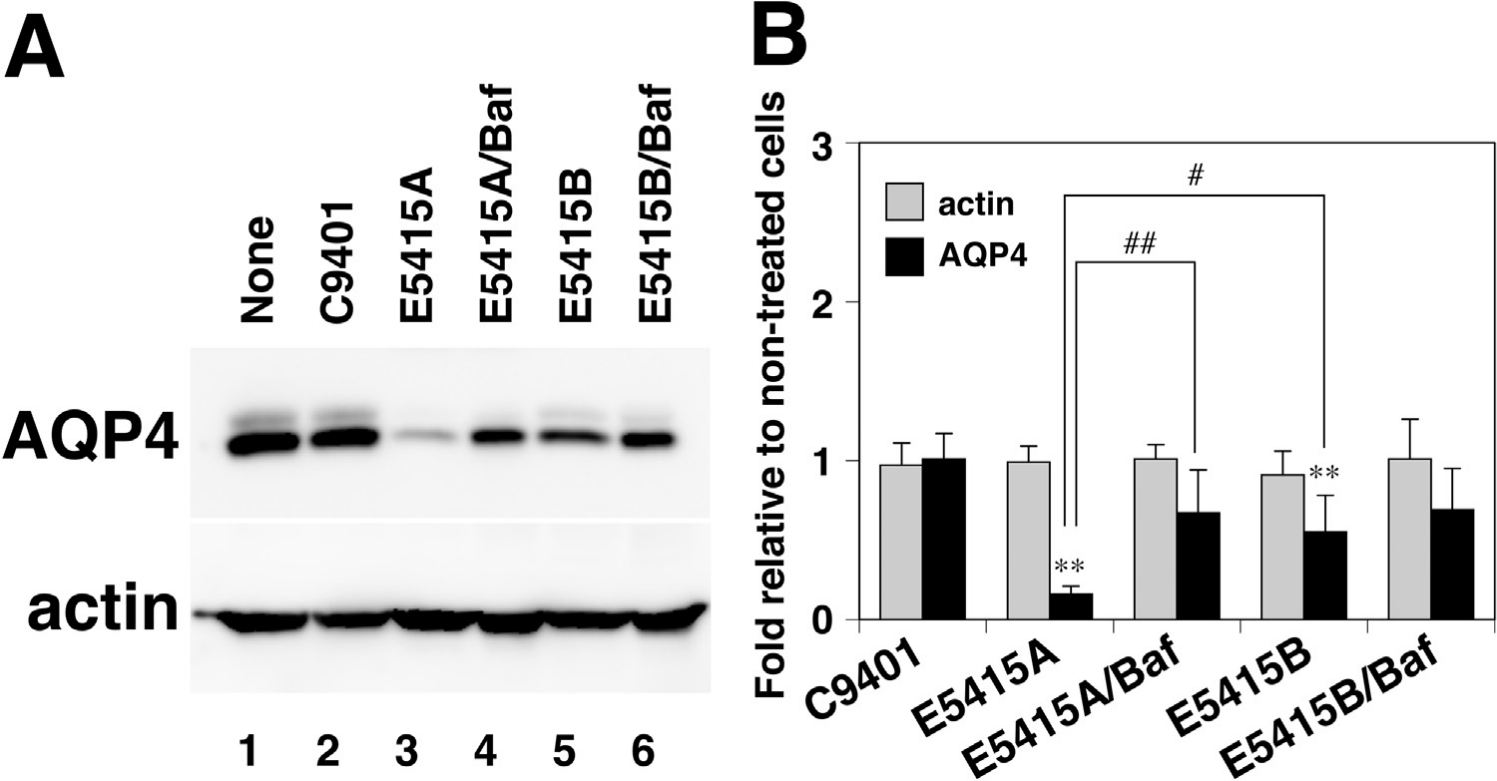
Effect of mAbs on levels of endogenous AQP4 in primary cultured astrocytes. (A) Representative immunoblots of lysates treated with 1 µg/ml of C9401 (lane 2), E5415A (lanes 3 and 4), or E5415B (lanes 5 and 6) in the absence (lanes 2, 3, and 5) or presence (lanes 4 and 6) of 500 nM bafilomycin A1 at 37 °C for 24 h using anti-AQP4 (upper panel) or anti-actin (lower panel) antibody. (B) Effect of E5415A or E5415B on levels of endogenous AQP4 (black column) and actin (grey column). Values are determined by Western blotting and estimated as fold relative to non-treated cells (A, lane 1). **(*P*<0.01), significant difference versus cells treated with C9401 alone; and ## (*P*<0.01) and # (*P*<0.05), significant difference between the groups as determined by the Tukey-Kramer method.

Using these mAbs, we first examined whether these antibodies enhance endocytosis of endogenous AQP4 in astrocytes. Both Alexa-Fluor-555-labeled E5415A and E5415B bound to astrocytes within 1 h (Fig. 1(A), (B), magenta). However, while fluorescence-labeled E5415A co-localized with Alexa-Fluor-488–conjugated transferrin in astrocytes during 3 h of incubation (Fig. 1(A)), fluorescence-labeled E5415B stayed on the cell surface up to 3 h of incubation, and little co-localization with Alexa-Fluor-488–conjugated transferrin was observed (Fig. 1(B)). Fluorescence-labeled E5415A was then detected in lysosomes as determined with LysoTracker Green DND-26 during 24 h of incubation (Fig. 1(C)). On the other hand, co-localization of E5415B with lysosomal markers was much less than that of E5415A (Fig. 1(D)). These observations demonstrated that E5415A enhanced endocytosis and transportation of endogenous AQP4 to lysosomes in astrocytes. Consistent with these observations, 24-h treatment with E5415A reduced endogenous AQP4 in astrocytes (Fig. 2(A), lane 3; Fig. 2(B)). Simultaneous treatment with bafilomycin A1 significantly restored the level of AQP4, indicating that AQP4 underwent lysosomal degradation (Fig. 2(A), lane 4; Fig. 2(B)). Although 24-h treatment of primary cultured astrocytes with E5415B also reduced endogenous AQP4, the level of the reduction induced by E5415B was less than that by E5415A (Fig. 2(A), lane 5; Fig. 2(B)).

### 3.3 E5415A promoted cluster formation of AQP4 on the cell surface of astrocytes prior to internalization

Although both E5415A and E5415B similarly bound to AQP4 expressed on the cell surface of astrocytes fixed before staining (Supplemental Fig. S8), E5415A enhanced endocytosis of AQP4 to a greater degree than did E5415B. This prompted us to examine what determined this difference between these two antibodies. Binding experiments with these mAbs using some CHO-cell clones expressing mAQP4 demonstrated that OAP-formation of AQP4 is advantageous for both E5415A and E5415B, providing them with a common binding site (Supplemental Fig. S2). OAP formation of AQP4 tightened the binding of both E5415A and E5415B to AQP4 by enhancing their re-association to AQP4 (Supplemental Fig. S3 and S4). In addition, E5415A had another binding site independent of OAP formation of AQP4 (the E5415A-specific binding site), to which E5415B hardly bound (Supplemental Fig. S2). E5415A strongly enhanced endocytosis of AQP4 in CHO cells expressing at least the M1 isoform (Supplemental Fig. S5-S7), suggesting that the binding of E5415A to the E5415A-specific site enhanced endocytosis of AQP4. E5415A induced complement-dependent cytotoxicity in CHO cells expressing M1 alone (Supplemental Fig. S9). This result strongly suggested that OAP-independent binding of E5415A to AQP4 promoted cluster formation of AQP4, since clustering of IgG as a result of that of AQP4 is necessary for multivalent binding of complement C1q. Phuan et al. had demonstrated that a recombinant NMO-IgG, rAb58, which binds to both M1 and M23 with similar affinity in a monovalent manner [15], induced no complement-dependent cytotoxicity (CDC) in cells expressing the M1 isoform alone [31]. This result supports our idea. Thus, we examined whether either antibody promoted clustering of AQP4 on the cell surfaces of astrocytes.

**Fig. 3:**
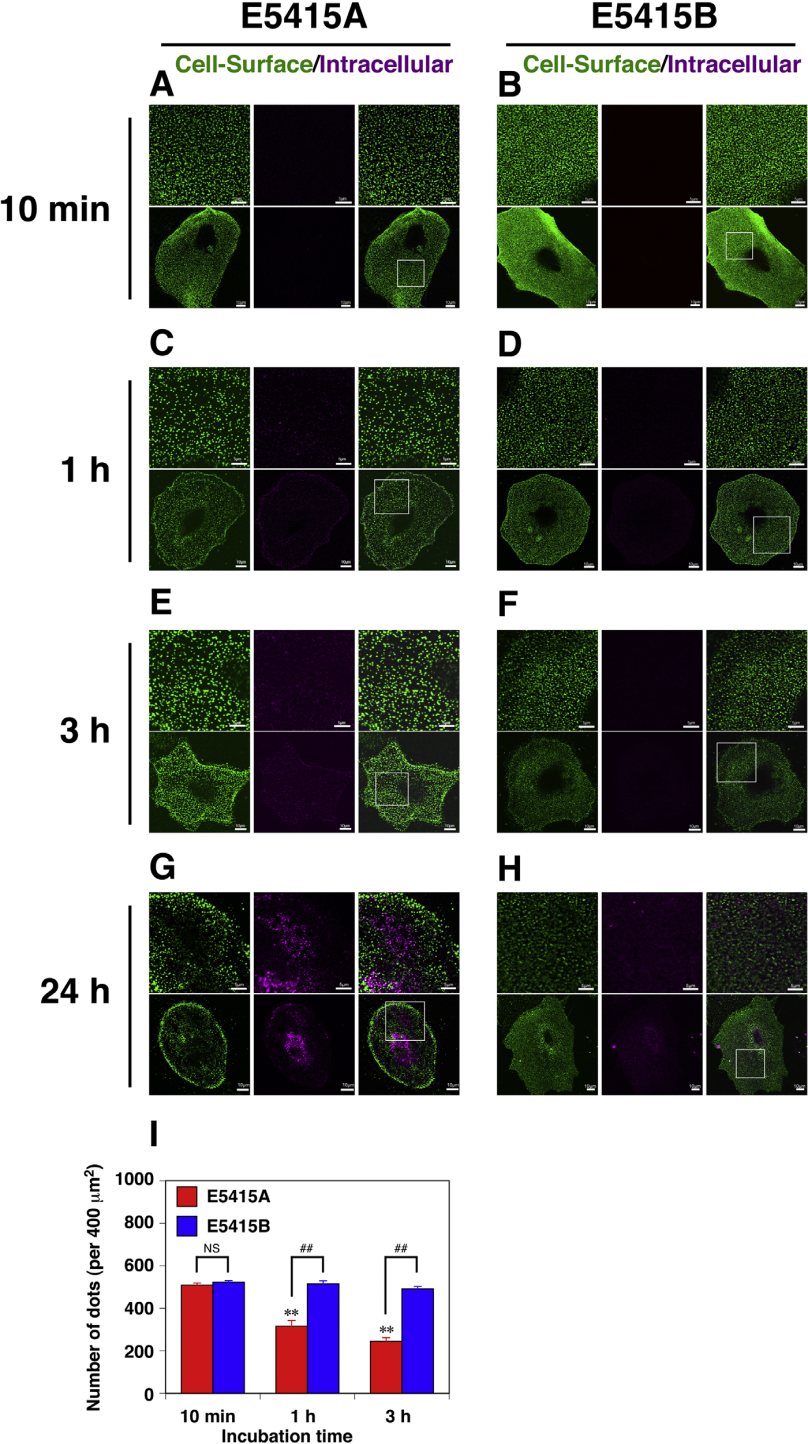
Cluster formation of AQP4 by binding of E5415A on an astrocytic membrane. Primary cultured astrocytes treated with E5415A (A, C, E, and G) or E5415B (B, D, F, and H) for 10 min (A and B), 1 h (C and D), or 3 h (E and F), or 24 h (G and H) at 37 °C were cooled to 4 °C and further treated with Alexa-Fluor-488-labeled anti-mouse IgG (green) at 4 °C for 1 h. Then cells were fixed with 4% PFA and permeabilized to detect intracellular mAb with Alexa-Fluor-555-labeled anti-mouse IgG (magenta). Bar=5 µm. The lower panels are global images of the upper panels. Magnified areas are indicated by white boxes. Bar=10 µm. (I) Quantification of the number of dots on the cell surface of primary cultured astrocytes incubated with E5415A (red bars) or E5415B (blue bars) followed by visualized with Alexa-Fluor-488-labeled anti-mouse IgG. Fifteen to twenty one images from five cells were taken in each experiment. Numbers of dots in 400 µm^2^ were counted using ImageJ2 (National Institute of Health, Bertesda, MD, http://imagej.nih.gov/ij/) software. Values are means±SD of four independent experiments. **(*P*<0.01), significant difference versus cells treated with E5415A for 10 min; ## (*P*<0.01), significant difference between the groups; and NS, not significant as determined by the Tukey-Kramer method.

Incubation of live astrocytes with E5415A resulted in binding of the antibody within 10 min (Fig. 3(A)), and in 1 h, coarse punctate distribution of the antibody on the cell surface, which is represented by significant reduction of number of dots in a field (Fig. 3(I)), was observed (Fig. 3(C), green). Intracellularly localized E5415A was not detected in astrocytes until after 24 h of incubation with this antibody, indicating that the reduction of the number of dots was not due to loss of AQP4 from the cell surface by its endocytosis (Fig. 3(A), (C), (E), (G), magenta). These results suggest that E5415A promotes cluster formation of AQP4 on the cell surface of astrocytes before internalization. Consistent with the result observed in astrocytes, E5415A also changed the smooth cell-surface staining pattern of CHO cells expressing M1 alone to a punctate one (Supplemental Fig. S10). On the other hand, intracellularly localized E5415B in astrocytes was not obvious up to 24 h of incubation (Fig. 3(B), (D), (F), (H), magenta). Although cell-surface E5415B (Fig. 3(B), (D), (F), (H), green) also showed punctate distribution, it was relatively fine throughout the incubation time as compared with E5415A (Fig. 3(I)). These observations support the notion that cluster formation of AQP4 by E5415A triggers endocytosis.

**Fig. 4:**
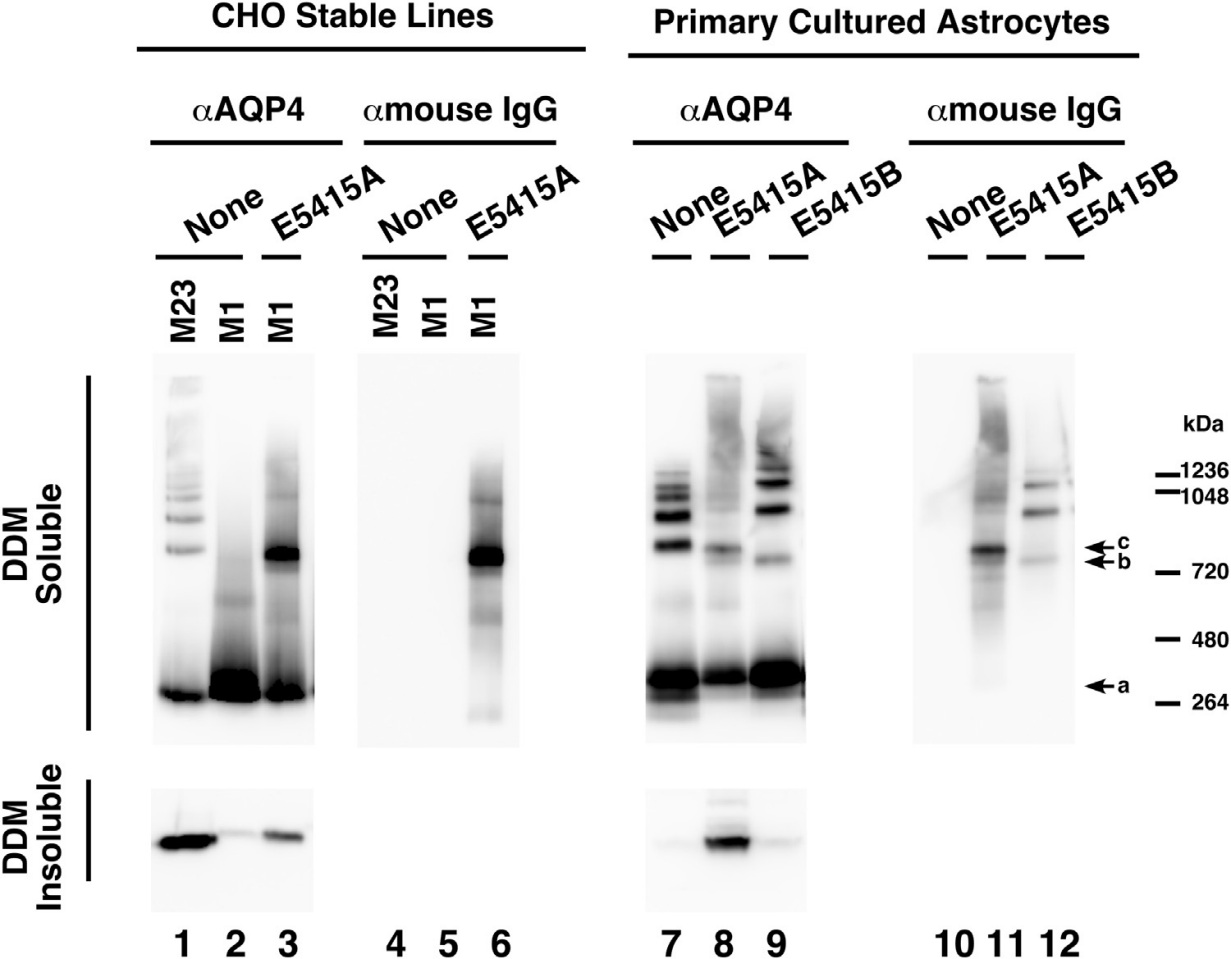
BN-PAGE analysis of complexes containing AQP4 in cells treated with mAbs. CHO cells stably expressing M23 alone (lanes 1 and 4), CHO cells stably expressing M1 alone (lanes 2, 3, 5, and 6), or primary cultured astrocytes (lanes 7–12) were incubated in the absence (lanes 1, 2, 4, 5, 7, and 10), or presence of 1.5 μg/ml of E5415A (lanes 3, 6, 8, and 11) or E5415B (lanes 9 and 12) for 3 h. DDM-soluble fractions (upper panels) and DDM-insoluble, SDS-soluble fractions (lower panels) were subjected to BN-PAGE and SDS-PAGE, respectively. Complexes containing AQP4 (lanes 1, 2, 3, 7, 8, and 9) and the mAbs (lanes 4, 5, 6, 10, 11, and 12) were detected with rabbit anti-AQP4 C-terminal domain antibody followed by HRP-labeled anti-rabbit IgG (α-AQP4) and HRP-labeled anti-mouse IgG (α-mouse IgG), respectively. A single AQP4 tetramer is indicated by arrow a. Complexes containing AQP4 and mouse IgG observed in cells treated with the mAbs for 3 h by Blue native PAGE are indicated by arrows b and c.

To further confirm the cluster formation of AQP4 by E5415A, we employed BN-PAGE of lysates solubilized in 10% DDM-containing buffer to see the size of complexes containing AQP4 tetramers. As previously reported [18], [31], when M1 alone was expressed in CHO cells, a single band corresponding to a single AQP4 tetramer was dominantly observed (Fig. 4, lane 2, indicated with arrow a). But in addition to the single tetramer, a ladder of larger complexes as well as a smear migrating slowly in the electrophoresis, which were derived from huge arrays, were observed in CHO cells expressing M23 alone (Fig. 4, lane 1). In addition, a significant level of AQP4 was detected in the DDM-insoluble, SDS-soluble fraction in cells expressing M23 alone (Fig. 4, lane 1, lower panel), whereas AQP4 in this fraction was much less in those expressing M1 alone (Fig. 4, lane 2, lower panel). When CHO cells expressing M1 alone were incubated with E5415A for 3 h, the single AQP4 tetramer was reduced (Fig. 4, lane 3, arrow a) and a strong signal migrating more slowly than the single AQP4 tetramer was observed (Fig. 4, lane 3, arrow c). This band was also detected with anti-mouse IgG, indicating that the band is a complex containing at least E5415A and AQP4 (Fig. 4, lane 6, arrow c). Interestingly, AQP4 in the DDM-insoluble fraction in E5415A-treated CHO cells expressing M1 alone increased (Fig. 4, lane 3), indicating that some population in AQP4-E5415A complexes becomes resistant to DDM in E5415A-treated CHO cells expressing M1 alone.

In the case of primary cultured astrocytes, in the absence of treatment with mAbs, a single AQP4 tetramer and a ladder with higher molecular masses were also observed; however, a smear like the one observed in CHO cells expressing M23 alone (Fig. 4, lane 1) was not apparent (Fig. 4, lane 7). When astrocytes were incubated with E5415A, the level of the single AQP4 tetramer was reduced (Fig. 4, lane 8, arrow a), and a band corresponding to the dominant AQP4-E5415A complex detected in E5415A-treated CHO cells expressing M1 alone was also observed (Fig. 4, lanes 8, 11, arrow c). Furthermore, the ladder became obscure, and instead, a long smear consisting of AQP4 and E5415A appeared. And AQP4 in the DDM-insoluble fraction increased. These observations indicated that binding of E5415A to endogenous AQP4 in astrocytes resulted in the formation of huge complexes of AQP4 and E5415A and that some of them became resistant to DDM, supporting the idea that E5415A promotes clustering of AQP4 on astrocyte surfaces. In contrast, when astrocytes were incubated with E5415B, the level of the single AQP4 tetramer was not altered, and equidistant upward shifts of the ladder were observed. In addition, the amount of AQP4 in the DDM-insoluble fraction in E5415B-treated cells was much less than that in E5415A-treated cells (Fig. 4, lanes 9, 8, respectively). Thus, it is highly likely that E5415B mainly bound to AQP4 located within arrays without cross-linking more than an array, which fails to contribute to further clustering of AQP4 or to the enhancement of endocytosis. Interestingly, a band containing AQP4 and mouse IgG (E5415B) migrating slightly faster than the dominant AQP4-E5415A complex detected in E5415A-treated CHO cells expressing M1 alone as well as in astrocytes was observed (Fig. 4, lanes 9, 12, arrow b), which was also detected as a minor band in E5415A-treated CHO cells expressing M1 alone and in astrocytes (Fig. 4, lanes 3, 6, 8, 11, arrow b).

Taken together, these two mAbs with different binding properties, and our analysis of the consequences of their binding to AQP4, clearly demonstrated that an anti-AQP4-ECDs antibody possessing an ability to form a large cluster of AQP4 by cross-linking two or more tetramers outside the AQP4 arrays enhances endocytosis and the subsequent lysosomal degradation of AQP4, which clarifies the controversial effects of a variety of NMO-IgGs on AQP4.

## Appendix A Transparency document

.

Transparency document Transparency document

.

Transparency document Transparency document

.

Transparency document Transparency document

.

Transparency document Transparency document

.

Transparency document Transparency document

.

Transparency document Transparency document

.

Transparency document Transparency document

.

Transparency document Transparency document

.

Transparency document Transparency document

.

Transparency document Transparency document

.

Transparency document Transparency document

.

Transparency document Transparency document

.

Transparency document Transparency document

## References

[1] Lennon V.A., Wingerchuk D.M., Kryzer T.J., Pittock S.J., Lucchinetti C.F., Fujihara K. (2004). A serum autoantibody marker of neuromyelitis optica: distinction from multiple sclerosis. Lancet.

[2] Lennon V.A., Kryzer T.J., Pittock S.J., Verkman A.S., Hinson S.R. (2005). IgG marker of optic-spinal multiple sclerosis binds to the aquaporin-4 water channel. J. Exp. Med..

[3] Fujihara K. (2011). Neuromyelitis optica and astrocytic damage in its pathogenesis. J. Neurol. Sci..

[4] Verkman A.S., Phuan P.W., Asavapanumas N., Tradtrantip L. (2013). Biology of AQP4 and anti-AQP4 antibody: therapeutic implications for NMO. Brain Pathol..

[5] Lucchinetti C.F., Guo Y., Popescu B.F., Fujihara K., Itoyama Y., Misu T. (2014). The pathology of an autoimmune astrocytopathy: lessons learned from neuromyelitis optica. Brain Pathol..

[6] Papadopoulos M.C., Bennett J.L., Verkman A.S. (2014). Treatment of neuromyelitis optica: state-of-the-art and emerging therapies. Nat. Rev. Neurol..

[7] Zekeridou A., Lennon V.A. (2015). Aquaporin-4 autoimmunity. Neurol. Neuroimmunol. Neuroinflamm..

[8] Hinson S.R., Roemer S.F., Lucchinetti C.F., Fryer J.P., Kryzer T.J., Chamberlain J.L. (2008). Aquaporin-4-binding autoantibodies in patients with neuromyelitis optica impair glutamate transport by down-regulating EAAT2. J. Exp. Med..

[9] Yang B., Brown D., Verkman A.S. (1996). The mercurial insensitive water channel (AQP-4) forms orthogonal arrays in stably transfected Chinese hamster ovary cells. J. Biol. Chem..

[10] Furman C.S., Gorelick-Feldman D.A., Davidson K.G., Yasumura T., Neely J.D., Agre P. (2003). Aquaporin-4 square array assembly: opposing actions of M1 and M23 isoforms. Proc. Natl. Acad. Sci. USA.

[11] Lu M., Lee M.D., Smith B.L., Jung J.S., Agre P., Verdijk M.A. (1996). The human AQP4 gene: definition of the locus encoding two water channel polypeptides in brain. Proc. Natl. Acad. Sci. USA.

[12] Suzuki H., Nishikawa K., Hiroaki Y., Fujiyoshi Y. (1778). Formation of aquaporin-4 arrays is inhibited by palmitoylation of N-terminal cysteine residues. Biochim. Biophys. Acta.

[13] Nicchia G.P., Mastrototaro M., Rossi A., Pisani F., Tortorella C., Ruggieri M. (2009). Aquaporin-4 orthogonal arrays of particles are the target for neuromyelitis optica autoantibodies. Glia.

[14] Pisani F., Mastrototaro M., Rossi A., Nicchia G.P., Tortorella C., Ruggieri M. (2011). Identification of two major conformational aquaporin-4 epitopes for neuromyelitis optica autoantibody binding. J. Biol. Chem..

[15] Crane J.M., Lam C., Rossi A., Gupta T., Bennett J.L., Verkman A.S. (2011). Binding affinity and specificity of neuromyelitis optica autoantibodies to aquaporin-4 M1/M23 isoforms and orthogonal arrays. J. Biol. Chem..

[16] Miyazaki K., Abe Y., Iwanari H., Suzuki Y., Kikuchi T., Ito T. (2013). Establishment of monoclonal antibodies against the extracellular domain that block binding of NMO-IgG to AQP4. J. Neuroimmunol..

[17] Pisani F., Sparaneo A., Tortorella C., Ruggieri M., Trojano M., Mola M.G. (2013). Aquaporin-4 autoantibodies in Neuromyelitis Optica: AQP4 isoform-dependent sensitivity and specificity. PLoS One.

[18] Owens G.P., Ritchie A., Rossi A., Schaller K., Wemlinger S., Schumann H. (2015). Mutagenesis of the aquaporin 4 extracellular domains defines restricted binding patterns of pathogenic neuromyelitis optica IgG. J. Biol. Chem..

[19] Hinson S.R., Pittock S.J., Lucchinetti C.F., Roemer S.F., Fryer J.P., Kryzer T.J. (2007). Pathogenic potential of IgG binding to water channel extracellular domain in neuromyelitis optica. Neurology.

[20] Hinson S.R., Romero M.F., Popescu B.F., Lucchinetti C.F., Fryer J.P., Wolburg H. (2012). Molecular outcomes of neuromyelitis optica (NMO)-IgG binding to aquaporin-4 in astrocytes. Proc. Natl. Acad. Sci. USA.

[21] Ratelade J., Bennett J.L., Verkman A.S. (2011). Evidence against cellular internalization in vivo of NMO-IgG, aquaporin-4, and excitatory amino acid transporter 2 in neuromyelitis optica. J. Biol. Chem..

[22] Vincent T., Saikali P., Cayrol R., Roth A.D., Bar-Or A., Prat A. (2008). Functional consequences of neuromyelitis optica-IgG astrocyte interactions on blood-brain barrier permeability and granulocyte recruitment. J. Immunol..

[23] Bennett J.L., Lam C., Kalluri S.R., Saikali P., Bautista K., Dupree C. (2009). Intrathecal pathogenic anti-aquaporin-4 antibodies in early neuromyelitis optica. Ann. Neurol..

[24] Saitoh R., Ohtomo T., Yamada Y., Kamada N., Nezu J., Kimura N. (2007). Viral envelope protein gp64 transgenic mouse facilitates the generation of monoclonal antibodies against exogenous membrane proteins displayed on baculovirus. J. Immunol. Methods.

[25] Ramadhanti J., Huang P., Kusano-Arai O., Iwanari H., Sakihama T., Misu T. (2013). A novel monoclonal antibody against the C-terminal region of aquaporin-4. Monoclon. Antib. Immunodiagn. Immunother..

[26] Ikeshima-Kataoka H., Abe Y., Abe T., Yasui M. (2013). Immunological function of aquaporin-4 in stab-wounded mouse brain in concert with a pro-inflammatory cytokine inducer, osteopontin. Mol. Cell. Neurosci..

[27] Abe Y., Ikeshima-Kataoka H., Goda W., Niikura T., Yasui M. (2012). An astrocyte-specific enhancer of the aquaporin-4 gene functions through a consensus sequence of POU transcription factors in concert with multiple upstream elements. J. Neurochem..

[28] Abe Y., Kita Y., Niikura T. (2008). Mammalian Gup1, a homolog of Saccharomyces cerevisiae glycerol uptake/transporter 1, acts as a negative regulator for N-terminal palmitoylation of Sonic hedgehog. FEBS J..

[29] Miyazaki-Komine K., Takai Y., Huang P., Kusano-Arai O., Iwanari H., Misu T. (2016). High avidity chimeric monoclonal antibodies against the extracellular domains of human aquaporin-4 competing with the neuromyelitis optica autoantibody, NMO-IgG. Br. J. Pharmacol..

[30] Kato J., Hayashi M.K., Aizu S., Yukutake Y., Takeda J., Yasui M. (2013). A general anaesthetic propofol inhibits aquaporin-4 in the presence of Zn^2+^. Biochem. J..

[31] Phuan P.-W., Ratelade J., Rossi A., Tradtrantip L., Verkman A.S. (2012). Complement-dependent cytotoxicity in neuromyelitis optica requires aquaporin-4 protein assembly in orthogonal arrays. J. Biol. Chem..

[32] Neely J.D., Christensen B.M., Nielsen S., Agre P. (1999). Heterotetrameric composition of aquaporin-4 water channels. Biochemistry.

[33] Crane J.M., Bennett J.L., Verkman A.S. (2009). Live cell analysis of aquaporin-4 M1/M23 interactions and regulated orthogonal array assembly in glial cells. J. Biol. Chem..

[34] Iorio R., Fryer J.P., Hinson S.R., Fallier-Becker P., Wolburg H., Pittock S.J. (2013). Astrocytic autoantibody of neuromyelitis optica (NMO-IgG) binds to aquaporin-4 extracellular loops, monomers and high order arrays. J. Autoimmun..

