# Supplementary material for: The binding property of a monoclonal antibody against the extracellular domains of aquaporin-4 directs aquaporin-4 toward endocytosis
Source: Biochem Biophys Rep. 2016 May 26;7:77–83. doi: 10.1016/j.bbrep.2016.05.017 (PMC5613303; doi:10.1016/j.bbrep.2016.05.017)
Supplement: Supplementary material [file mmc1.pdf]

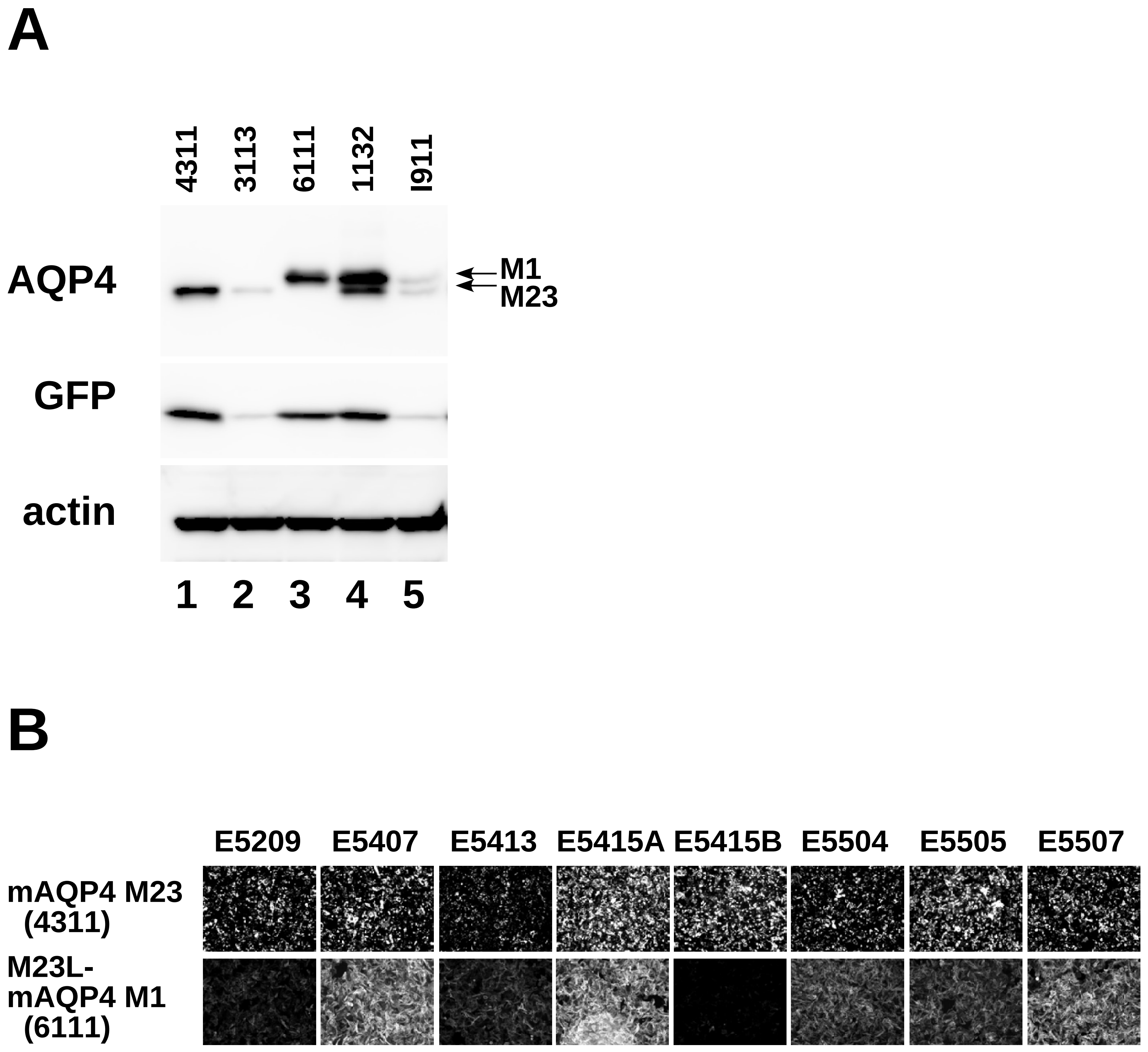

Supplemental Fig. S1. Establishment of stable CHO-cell clones expressing mAQP4 and mAbs against the extracellular domains of mAQP4.

(A) Establishment of stable CHO-cell clones expressing mAQP4. Expression of AQP4, EGFP, and actin in lysates (30 μg) from clones expressing mAQP4 M23 alone (clones 4311 and 3113, lanes 1 and 2), mAQP4 M1 alone (clone 6111, lane 3), and mAQP4 M1 and M23 (clones 1132 and I911, lanes 4, and 5) was determined by Western blotting. (B) Screening of monoclonal antibodies against the extracellular domains of mAQP4. Hybridoma culture supernatants were diluted 10-fold and subjected to immunofluorescent staining using CHO cells expressing M23 alone (mAQP4 M23, 4311 cells) and M1 alone (M23L-mAQP4 M1, 6111 cells). E5415A and E5415B were chosen as clones recognizing both M1 and M23 and M23 only, respectively.

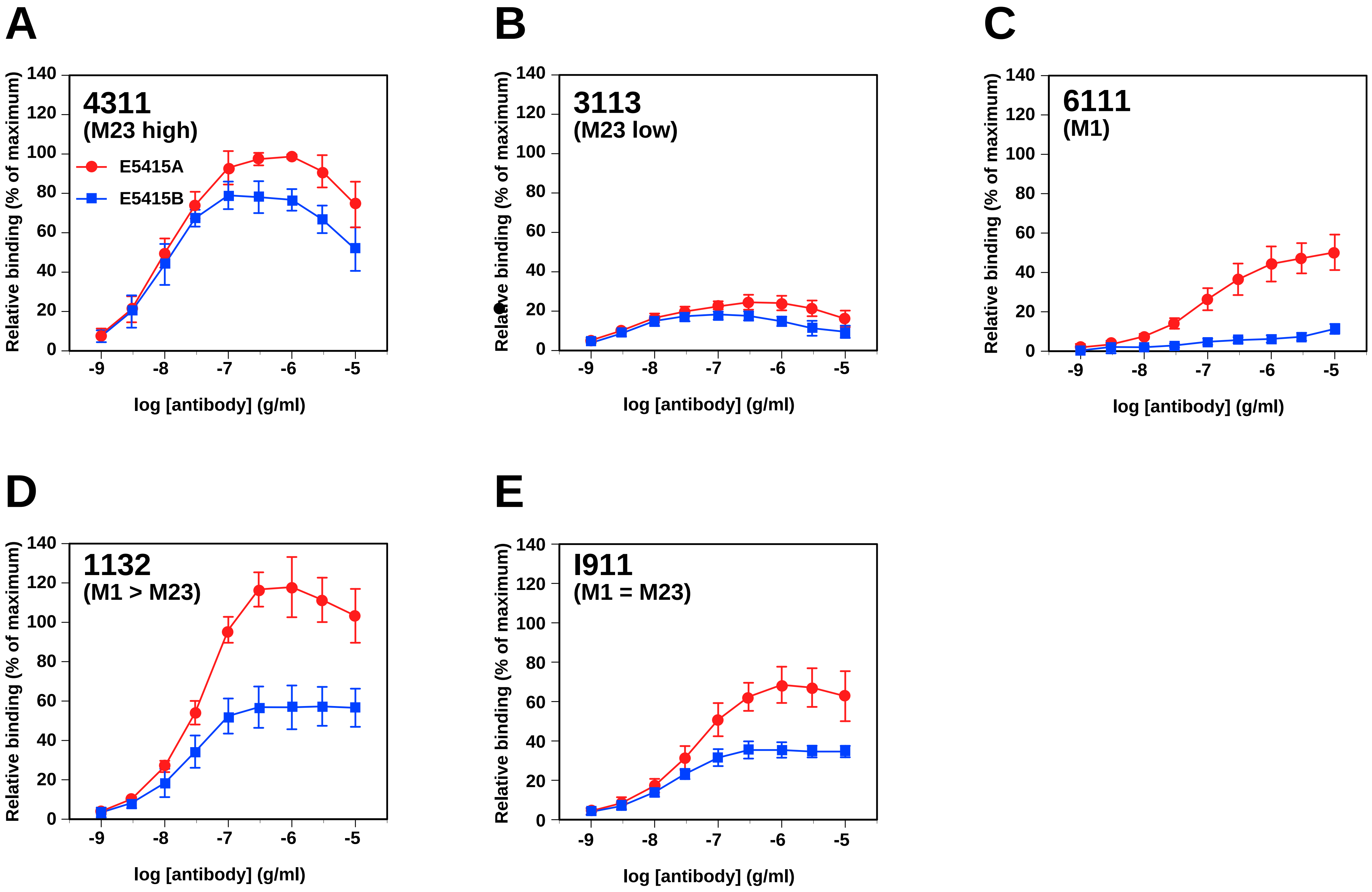

Supplemental Fig. S2. Saturation binding experiments of mAbs against the ECDs of mAQP4 expressed on stable CHO-cell clones determined by ELISA.

CHO cells stably expressing M23 alone (A and B), M1 alone (C), and both M1 and M23 (D, and E) of mAQP4 were incubated with either E5415A (red closed circles) or E5415B (blue closed squares) at 4°C overnight. Bound antibodies were measured by ELISA and estimated as the percent of maximal binding of E5415A to CHO-cell clone 4311, a line stably expressing high levels of mAQP4 M23 alone (A). Values are represented as means ± SD of 6-8 independent experiments performed in duplicate.

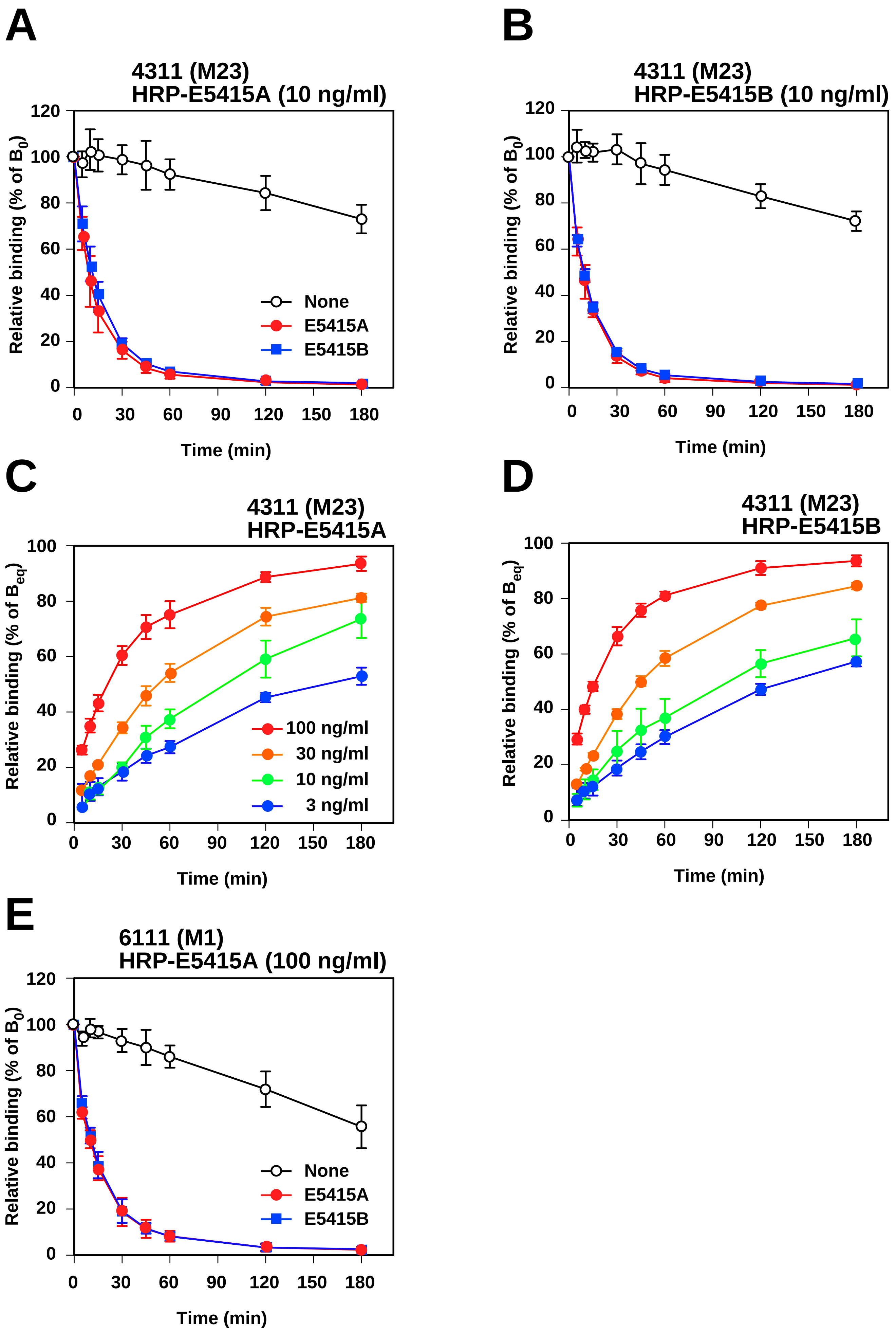

Supplemental Fig. S3. Kinetic experiments of mAbs against the ECDs of mAQP4 expressed on stable CHO-cell clones determined by ELISA.

(A, B) Determination of dissociation rate constants of HRP-labeled E5415A and E5415B from CHO cells expressing mAQP4 M23 (clone 4311). CHO cells expressing mAQP4 M23 (4311 cells) were incubated with HRP-labeled E5415A (A) or E5415B (B) (10 ng/ml) at 4˚C overnight. Then the dissociation was initiated at room temperature by dilution (black open circles), or by addition of an excess (10 µg/ml) of unlabeled E5415A (red closed circles) or unlabeled E5415B (blue closed squares). Bound HRP-labeled mAbs were determined by ELISA and estimated as percent of binding of each HRP-labeled mAb at the initiation of the dissociation (B_0). Values are means ± SD of four independent experiments performed in duplicate. (C, D) Determination of association rate constants of HRP-labeled E5415A (C) and E5415B (D) to CHO cells expressing mAQP4 M23 (clone 4311). 4311 cells were incubated with HRP-labeled E5415A or E5415B at concentrations of 3 ng/ml (blue circles), 10 ng/ml (green circles), 30 ng/ml (orange circles), and 100 ng/ml (red circles) at room temperature for the indicated time. Bound HRP-labeled mAbs were determined by ELISA and estimated as percent of the equilibrium binding of each HRP-labeled mAb (B_{eq}). Values are means ± SD of four independent experiments performed in duplicate. (E) Determination of dissociation rate constants of HRP-labeled E5415A from CHO cells expressing mAQP4 M1 alone (clone 6111). 6111 cells were incubated with HRP-labeled E5415A (100 ng/ml) at 4˚C overnight. Then the dissociation was initiated at room temperature by dilution (black open circles), or by addition of an excess (100 µg/ml) of unlabeled E5415A (red closed circles) or unlabeled E5415B (blue closed squares). Bound HRP-labeled E5415A was determined by ELISA and estimated as percent of its binding at the initiation of the dissociation (B_0). Values are means ± SD of five independent experiments performed in duplicate.

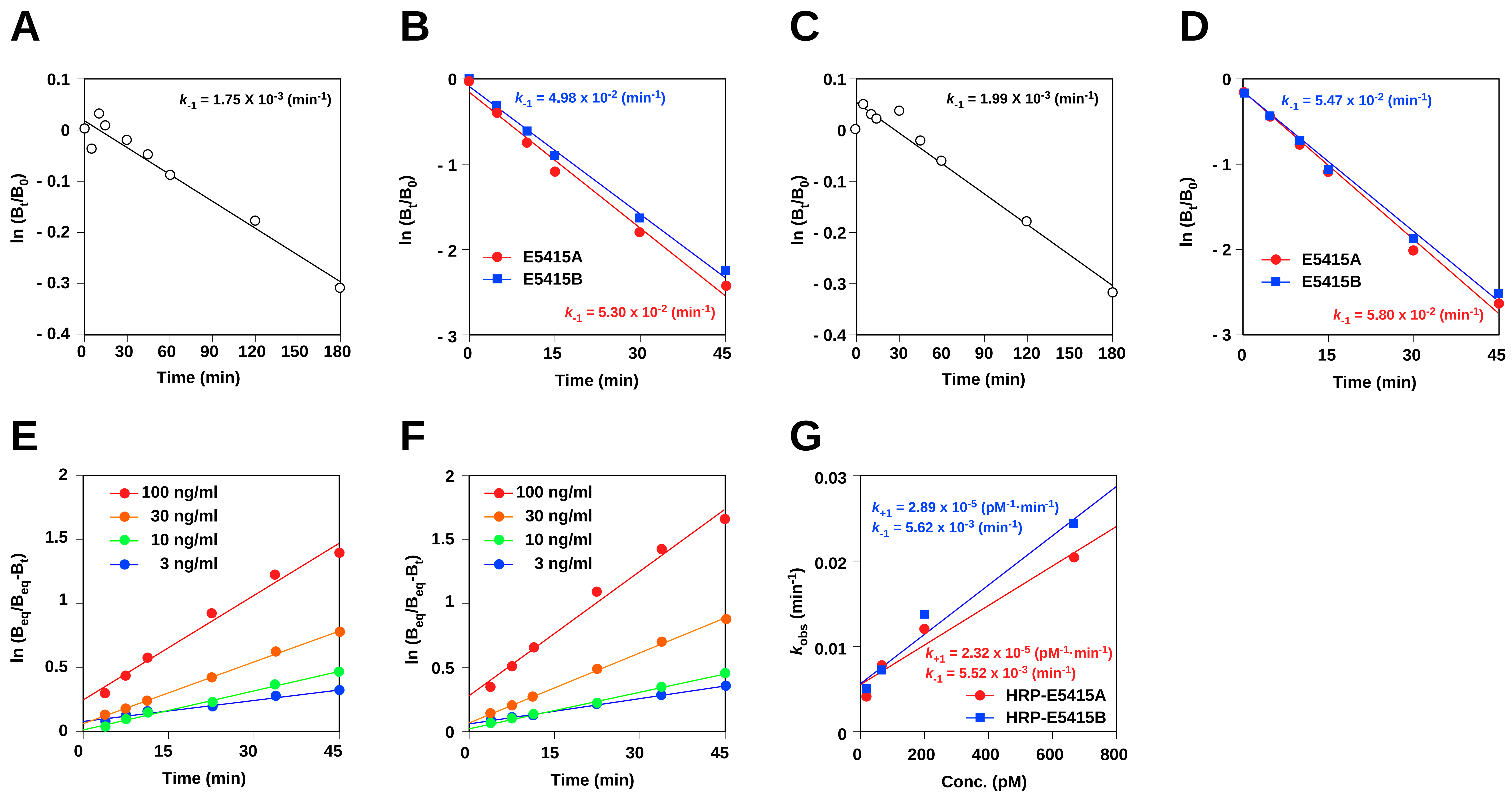

Supplemental Fig. S4. Kinetic experiment of mAbs against the ECDs of mAQP4.
(A-D) Determination of the dissociation rate constants (k_{-1}) of HRP-labeled E5415A and E5415B from CHO cells expressing mAQP4 M23. HRP-labeled E5415A (A, B) or E5415B (C, D) (10 ng/ml) were incubated with CHO cells expressing mAQP4 M23 (4311 cells) at 4˚C overnight. Then the dissociation was initiated at room temperature by dilution (A and C, black open circles), or by addition of an excess (10 µg/ml) of unlabeled E5415A (B and D, red closed circles) or unlabeled E5415B (B and D, blue closed squares). Bound HRP-labeled mAbs were determined by ELISA. k_{-1} values were obtained by plotting $\ln(B_t/B_0)$ versus the dissociation time based on the results of Fig. 3A and B. (E, F) Determination of the observed onset rate constant (k_{obs}) of HRP-labeled E5415A and E5415B against CHO cells expressing mAQP4 M23. HRP-labeled E5415A (E) and E5415B (F) at concentrations of 3 ng/ml (blue circles), 10 ng/ml (green circles), 30 ng/ml (orange circles), and 100 ng/ml (red circles) were incubated with 4311 cells at room temperature for indicated time, and bound HRP-labeled mAbs were determined by ELISA. k_{obs} values were obtained by plotting $\ln(B_{eq}/(B_{eq} - B_t))$ versus the association time based on the results of Fig. 3C and D. (G) Determination of the association rate constant (k_{+1}) of HRP-labeled E5415A (red closed circles) and E5415B (blue closed circles) to CHO cells expressing mAQP4 M23. k_{+1} values were obtained by plotting k_{obs} versus concentration of the mAbs based on the result of Supplemental Fig. S3E and F. Abbreviations: B_0, binding at the start of dissociation; B_t, binding at time t; B_{eq}, equilibrium binding; k_{-1}, dissociation rate constant; k_{+1}, association rate constant; k_{obs}, observed onset rate constant; t, time.

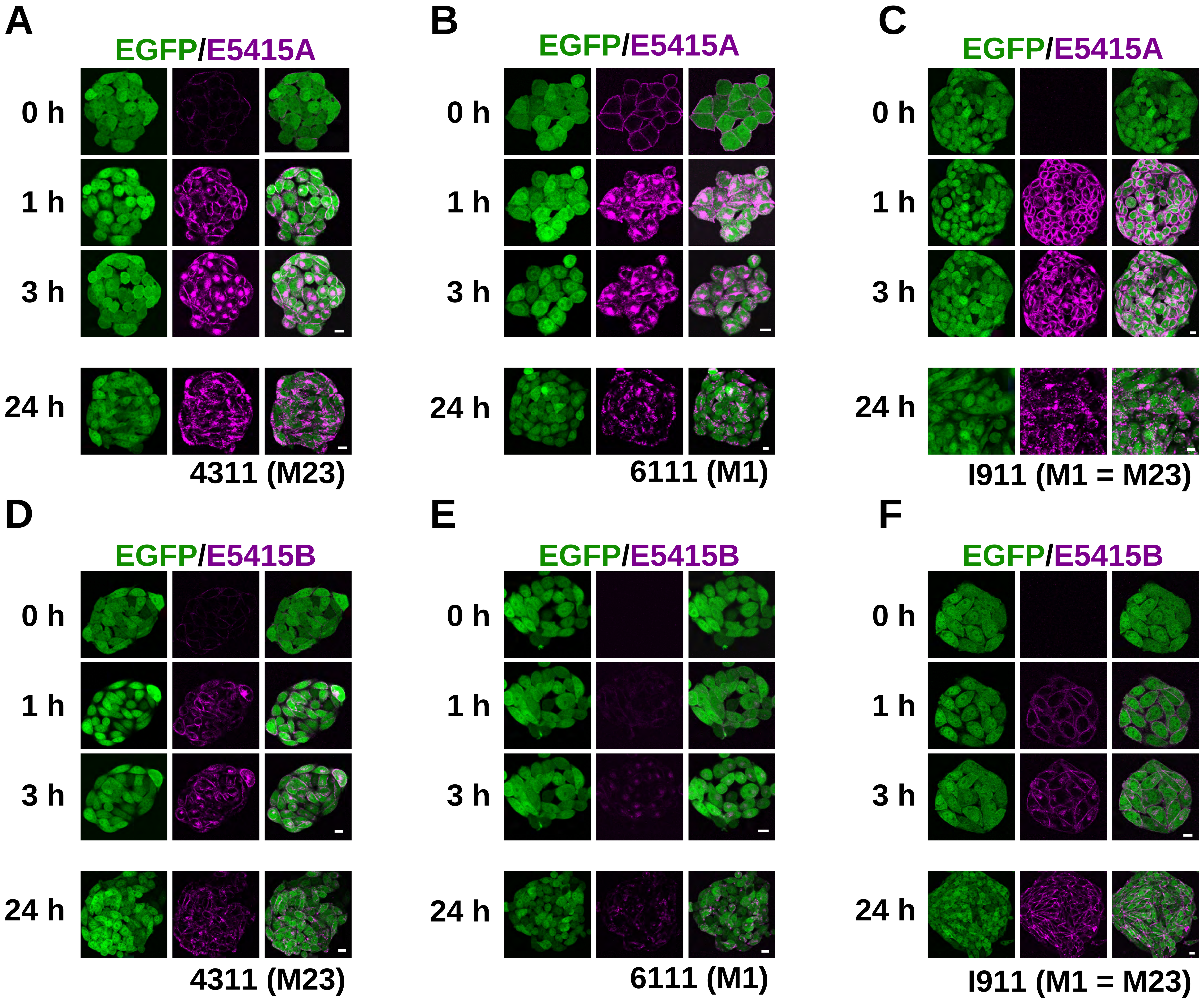

Supplemental Fig. S5. Live imaging of fluorescence-labeled mAbs in CHO-cell clones stably expressing mAQP4.
CHO-cell clones expressing mAQP4 M23 alone (A and D, clone 4311), M1 alone (B and E, clone 6111) and both M1 and M23 (C and F, clone I911) were incubated with 2 μg/ml of Alexa-Fluor-555-labeled E5415A (A, B and C) or E5415B (D, E, and F) at 37˚C for 3 h under observation with a confocal microscope, or for 24 h before observation with the microscope. EGFP expressed in each clone and bound labeled antibodies are represented in green and magenta, respectively. Bar = 10 μm.

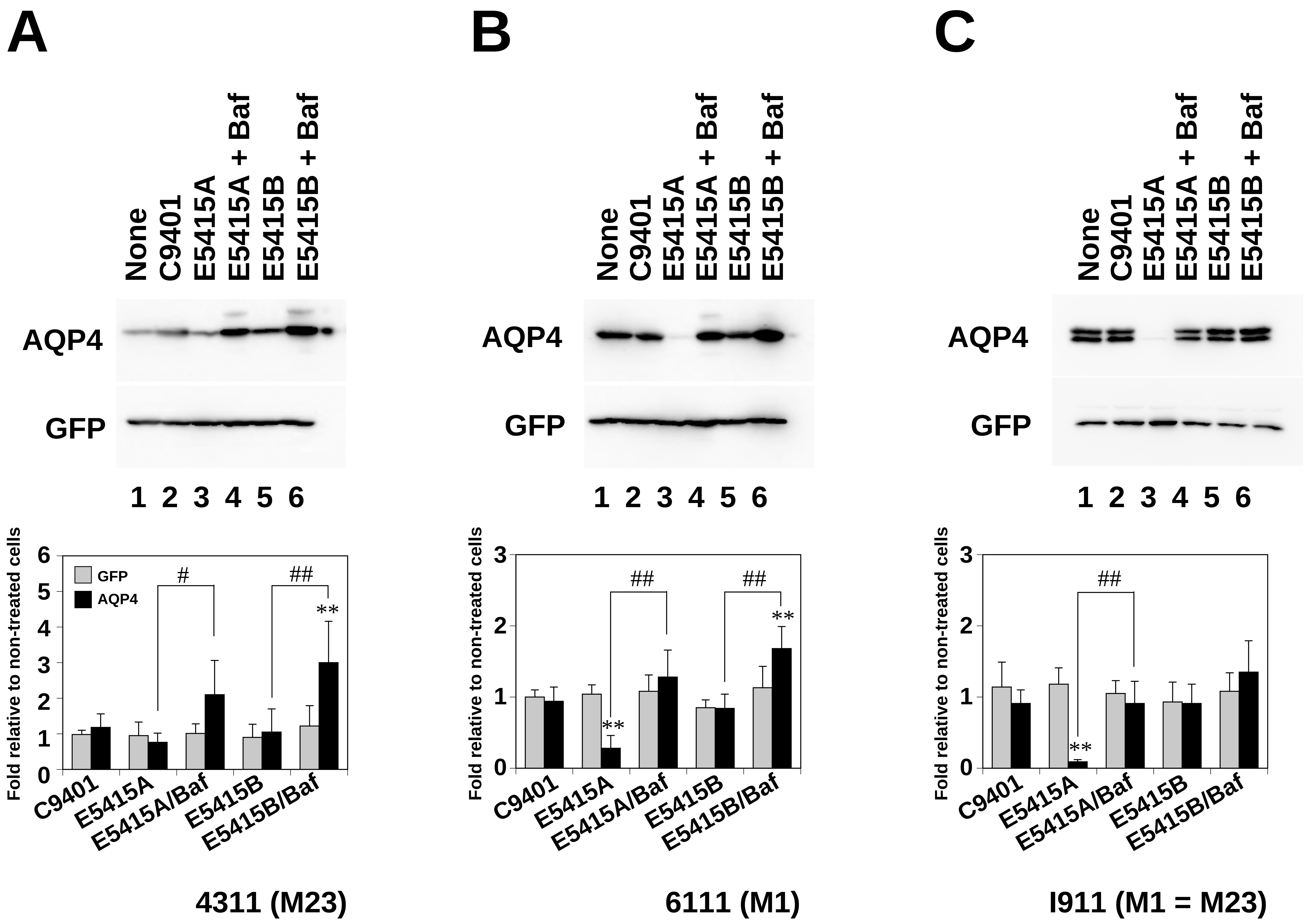

Supplmental Fig. S6. Effect of mAbs on levels of mAQP4 protein expressed in CHO-cell clones.
CHO cells expressing either M23 alone (A, 4311), M1 alone (B, 6111), or both M1 and M23 (C, I911) were treated with 1 μg/ml of E5415A (lanes 3 and 4), E5415B (lanes 5 and 6), or hAQP4-specific C9401 (lane 2) in the absence (lanes 2, 3, and 5) or presence (lanes 4 and 6) of 100 nM bafilomycin A1 at 37˚C for 24 h. Amounts of EGFP (grey column) and AQP4 (black column) were determined by Western blotting and estimated as fold relative to non-treated cells (lane 1). Values are represented as means ± SD of 6-7 independent experiments. ** (P < 0.01), significant difference versus cells treated with C9401 alone; and ## (P < 0.01) and # (P < 0.05), significant difference between with and without bafilomycin A1 treatment as determined by the Tukey-Kramer method.

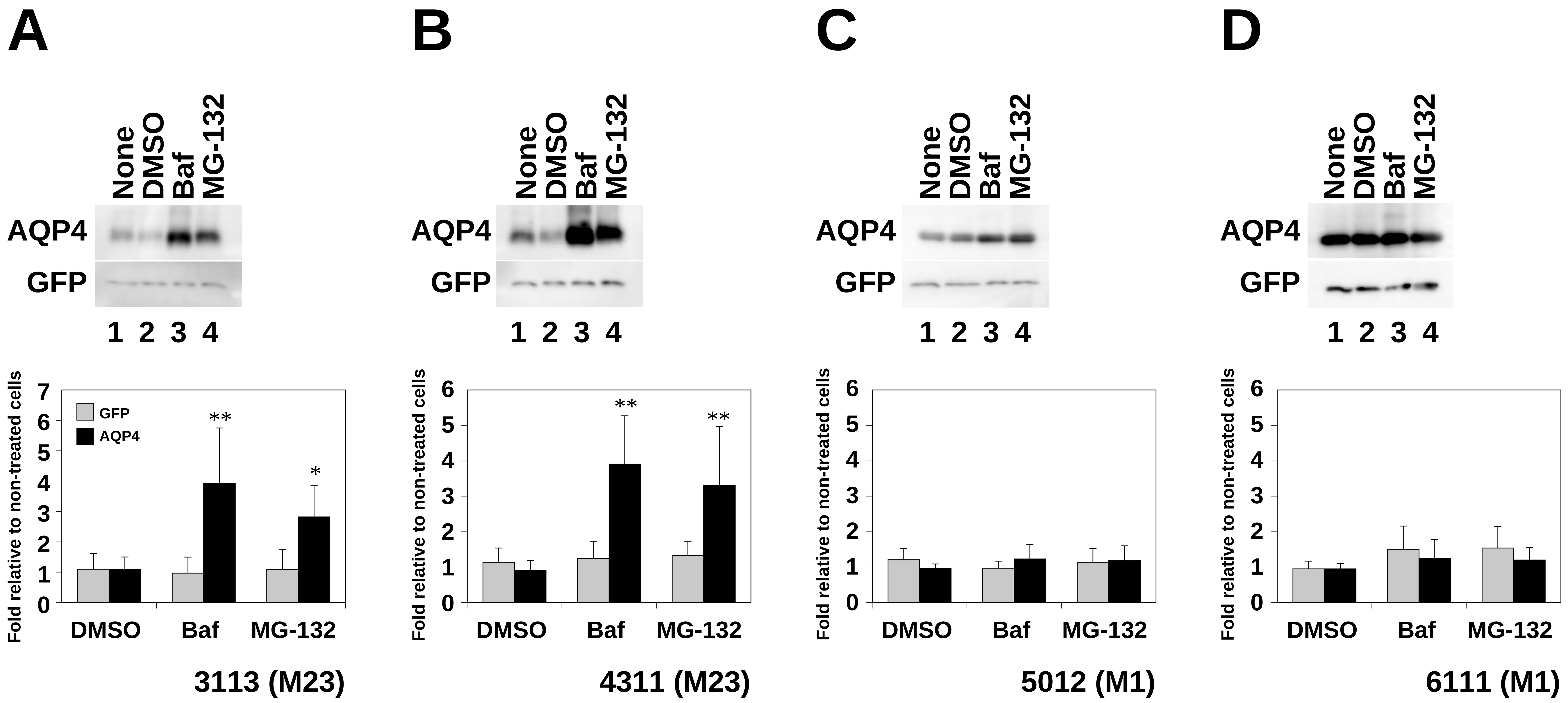

Supplemental Fig. S7. Effect of inhibition of protein degradation pathways on level of mAQP4 protein expressed in CHO-cell clones.

Cells expressing either M23 alone (A and B) or M1 alone (C and D) were treated with either 100 nM Bafilomycin A1 (lane 3, Baf), 1 mM MG-132 (lane 4), or vehicle control (lane 2, DMSO) at 37˚C for 24 h. Amounts of GFP (grey column) and AQP4 (black column) were determined by Western blotting and estimated as fold relative to non-treated cells (lane 1). Values are represented as means ± SD of 6-10 independent experiments. ** ($P < 0.01$) and * ($P < 0.05$), significant difference versus cells treated with DMSO as determined by the Tukey-Kramer method.

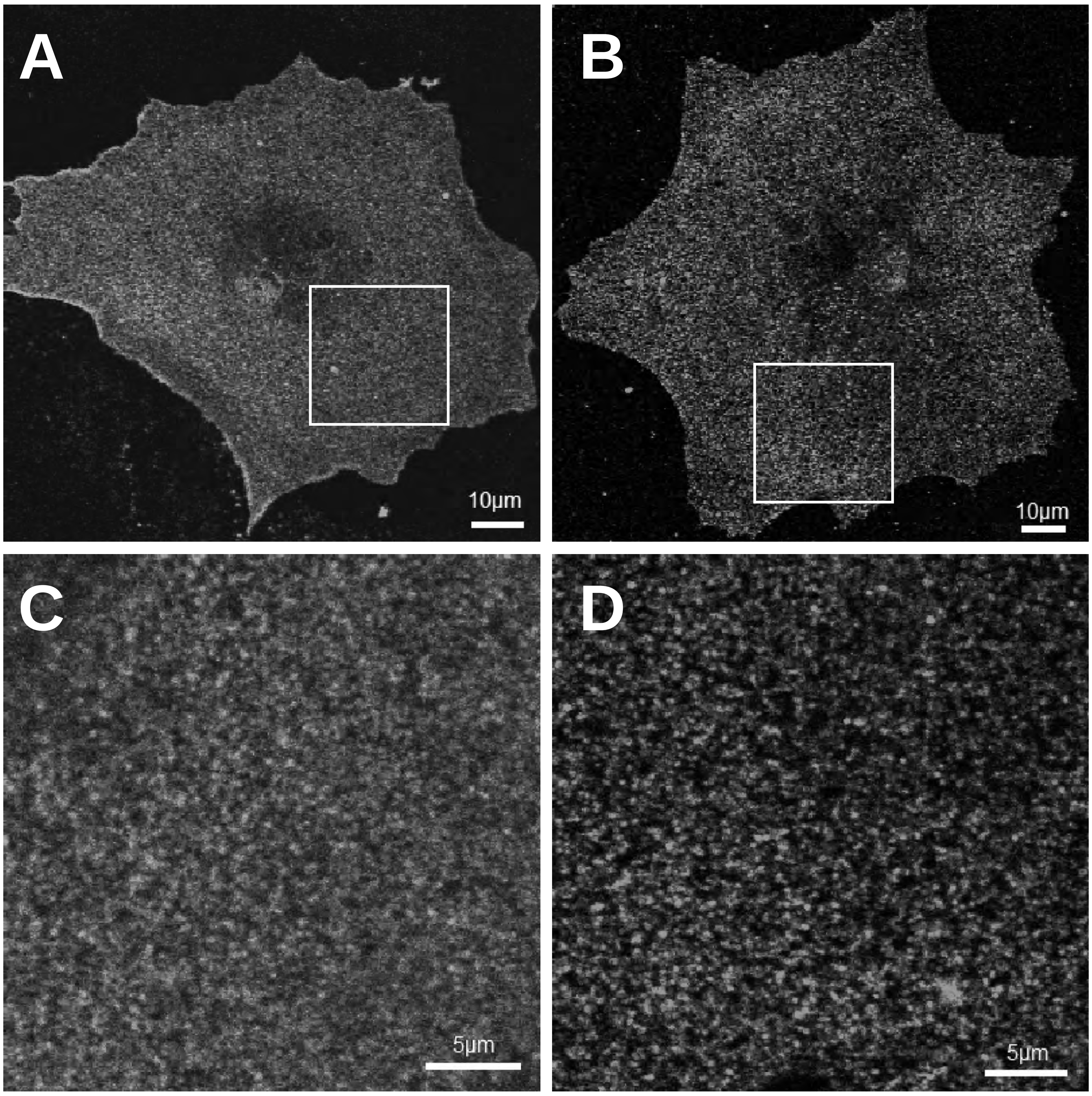

Supplemental Fig. S8. Immunofluorescent staining of AQP4 expressed in primary cultured astrocytes.

Fixed astrocytes were incubated with E5415A (A) or E5415B (C) and visualized with Alexa-Fluor-488-labeled anti-mouse IgG. Bar = 10 μm. Magnified images of the areas indicated by white boxes in (A) and (C) are shown in (B) and (D), respectively. Bar = 5 μm.

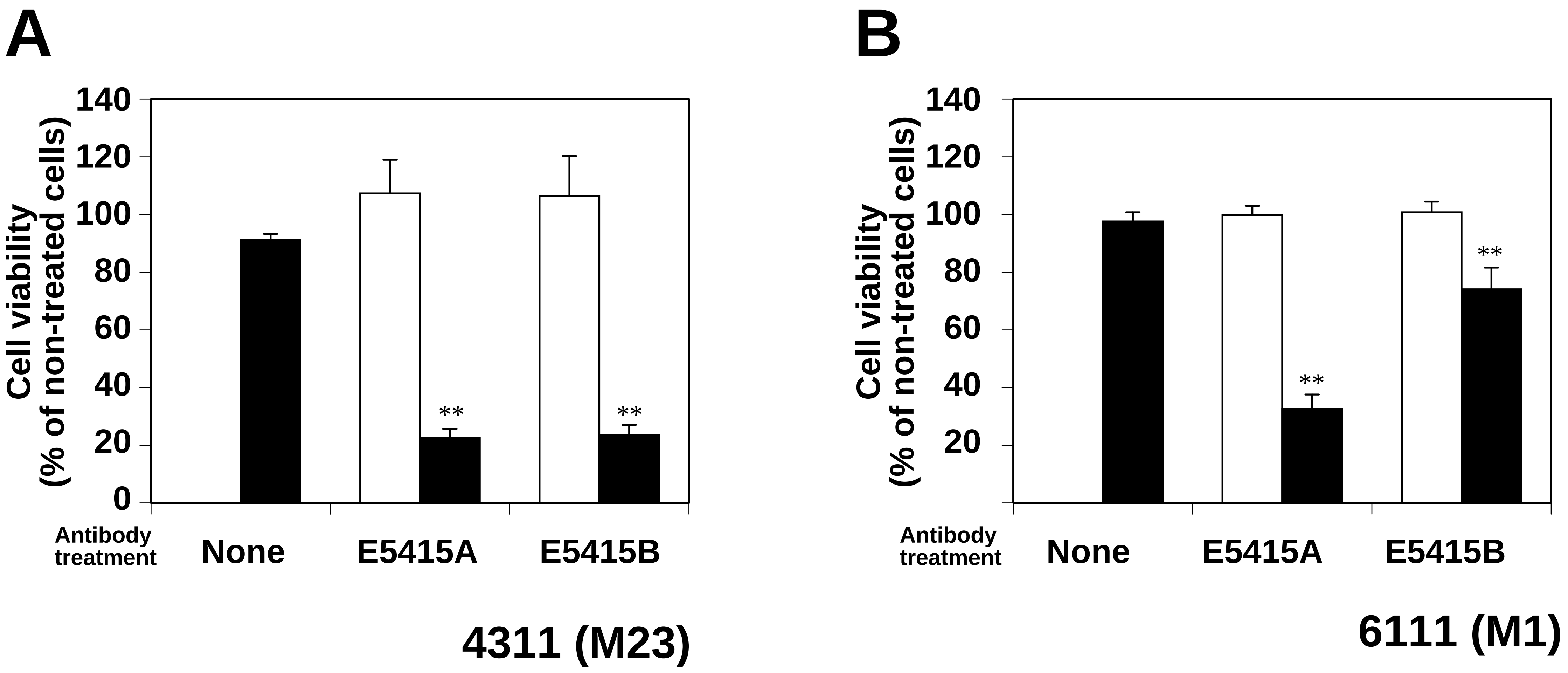

Supplemental Fig. S9. Complement-dependent cytotoxic effect of mAbs on CHO-cell clones expressing mAQP4.

E5415A or E5415B (2 μg/ml) were added to CHO cells expressing M23 (A) or M1 alone (B) at 37˚C for 90 min with (solid bars) or without (open bars) rabbit complement (2%). Cell viabilities were estimated as a percent of non-treated cells. Values are mean ± SD of 5-9 independent experiments. ** ($P < 0.01$), significantly different from cells without complement as determined by the Tukey-Kramer method.

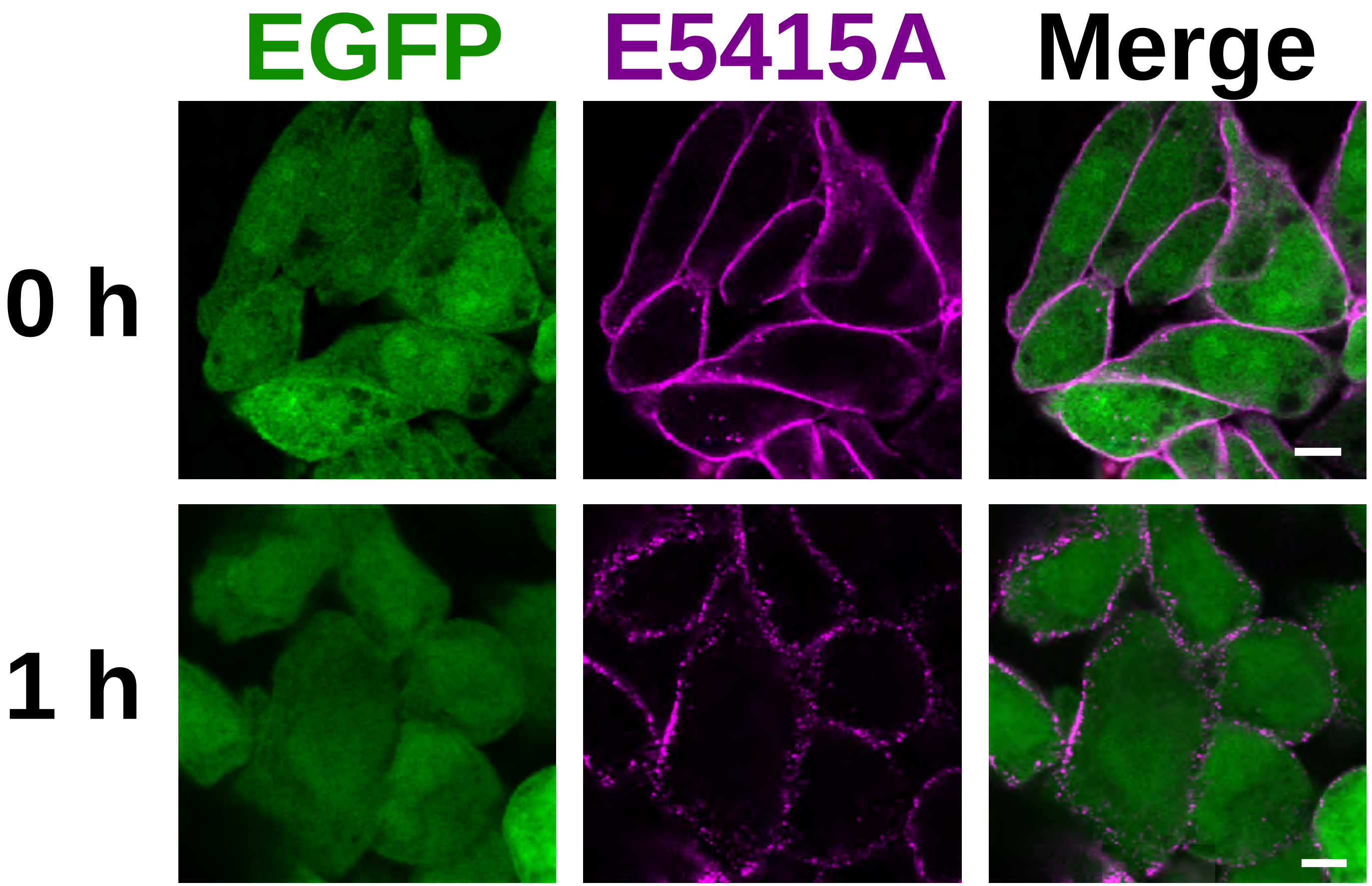

Supplemental Fig. S10. Cluster formation of AQP4 by binding of E5415A on CHO cells expressing M1 alone. Fixed CHO cell-clone expressing mAQP4 M1 (0 hr) were incubated with E5415A and visualized with Alexa-Fluor-555-labeled anti mouse IgG without permeabilization. Live cells incubated with E5415A for 1 h at 37˚C were cooled to 4˚C and further treated with Alexa-Fluor-555-labeled anti-mouse IgG at 4˚C for 1 h (1 h). Then cells were fixed with 4% PFA. EGFP expressed in the cells and bound antibody are represeneted in green and magenta, respectively. Bar = 5 μm

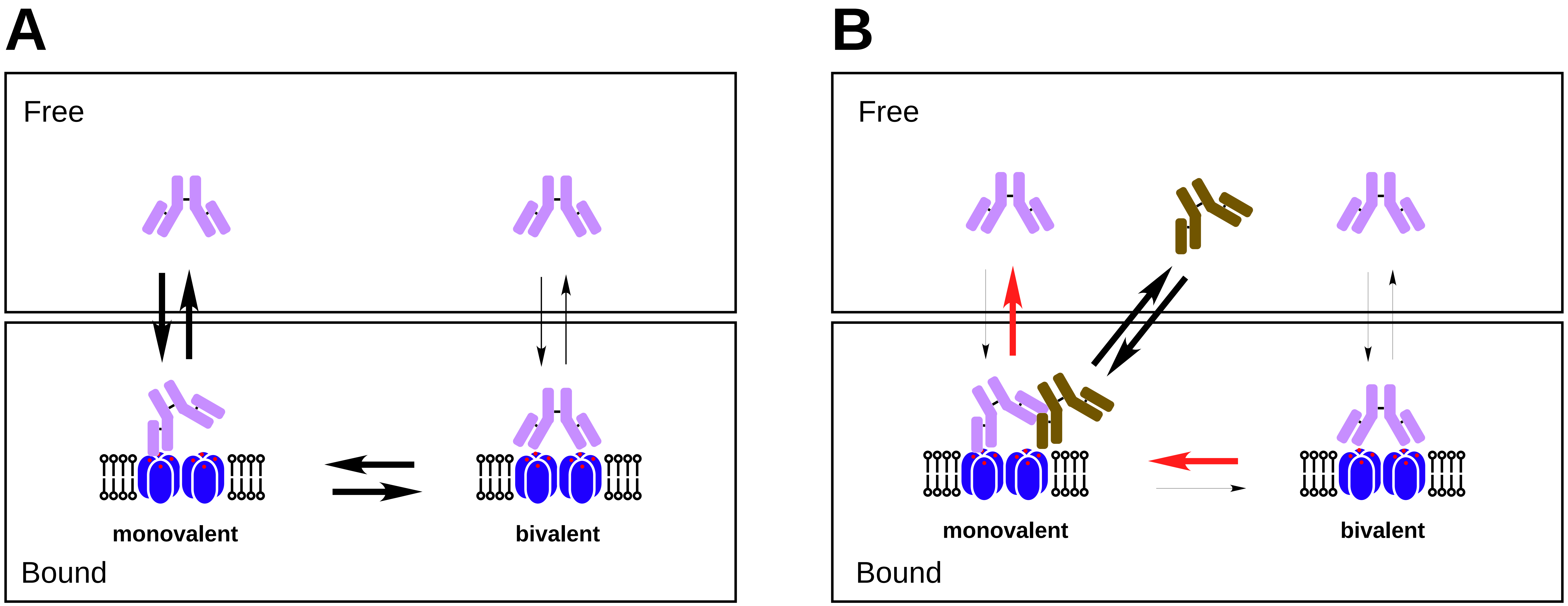

Supplemental Fig. S11. Schematic illustrations of a model for binding of E5415A and E5415B to AQP4.
Binding of E5415A and E5415B to AQP4 incorporated into OAPs in the absence (A) or presence (B) of competitors is shown. The mAbs and competitive antibodies are indicated in purple and brown, respectively. AQP4 tetramers integrated into a lipid bilayer and epitopes for the mAbs are represented as blue columns and red dots, respectively. Antibodies in free and bound states are indicated with boxes.
