# Supplementary material for: The binding property of a monoclonal antibody against the extracellular domains of aquaporin-4 directs aquaporin-4 toward endocytosis
Source: Biochem Biophys Rep. 2016 May 26;7:77–83. doi: 10.1016/j.bbrep.2016.05.017 (PMC5613303; doi:10.1016/j.bbrep.2016.05.017)
Supplement: Supplementary material [file mmc2.docx]

**Material and methods**

*Plasmid construction*

cDNAs encoding mAQP4 M1 and M23 were inserted into a pIRES2-EGFP vector (Clontech Laboratories, Mountain View, CA, USA), in which a unique AflII site had been modified to an EcoRI by linker ligation, resulting in pmAQP4-M1-IRES-EGFP and pmAQP4-M23-IRES-EGFP, respectively [1]. To avoid expression of the M23 isoform from an M1 transcript due to a leaky scanning mechanism [2], an M23L mutation of mAQP4 M1 isoform was introduced by a QuickChange site-directed mutagenesis kit (Agilent Technologies, Santa Clara, CA, USA) using primers 5’- CTGCAGTAGAGAGAGCATCCTGGTGGCTTTC-3’ and 5’-GAAAGCCACCAGGATGCTCTCTCTACTGCAG-3’ (pM23L-mAQP4-M1-IRES-EGFP). To establish CHO-cell clones stably expressing AQP4, each vector was linearized with EcoRI before transfection.

*Establishment of stable cell lines*

CHO cells maintained in Ham’s F12 medium supplemented with 10% fetal bovine serum, 50 units/ml penicillin, and 50 µg/ml streptomycin were seeded onto 35-mm dishes at a density of 2 × 10^5^ cells/dish and cultured for 24 h. Cells were then transfected with each linearized plasmid using Lipofectamine and Plus reagents (Life Technologies, Carlsbad, CA, USA) according to the manufacturer’s instructions. Two days after transfection, cells were trypsinized and reseeded onto ten 10-cm dishes in medium containing G418 (500 µg/ml, Nacalai Tesque, Inc., Kyoto, Japan). Approximately 10 days after selection with G418, several colonies positive for fluorescence of EGFP were harvested. After amplification and confirmation of AQP4 expression by Western blotting, several single-cell clones were obtained by limiting dilution (Supplemental Fig. S1A).

*Development of mAbs against the ECDs of mAQP4*

The eight clones (Supplemental Fig. S1B) were divided into three groups: three clones (IgG2a) recognizing both M1 and M23 isoforms of mAQP4 (E5407, E5415A, and E5507); four clones (IgG2a) preferentially recognizing the M23 isoform of mAQP4 (E5209, E5415B, E5504, and E5505); and one clone (IgG3) only weakly recognizing both M1 and M23 isoforms (E5413), by immunofluorescent staining using a CHO-cell clones expressing M23L-mAQP4 M1 (clone 6111, Supplemental Fig. S1A, lane 3) and expressing mAQP4 M23 (clone 4311, Supplemental Fig. S1A, lane 2).

*ELISA*

E5415A and E5415B were labeled with horseradish peroxidase (HRP) using Peroxidase Labeling Kit-NH_2_ (Dojindo Laboratories, Kumamoto, Japan).

CHO cells stably expressing AQP4 were seeded onto 96-well plates at a density of 1 × 10^5^ cells/well. Twenty-four hours later, cells were fixed with 4% PFA at 4˚C overnight. Fixed cells were washed twice with PBS and blocked with 40% Block Ace (DS Pharma Biomedical Co., Ltd. Osaka, Japan) for 1 h.

For saturation binding experiments, various concentrations of antibodies in 40% Block Ace were added to the 96-well plates and incubated at 4˚C overnight. Bound antibodies were detected by incubation with HRP-conjugated anti-mouse antibody (1:8000, Sigma, St Louis, MO) at room temperature for 1 h. After washing five times with PBS, signals were visualized by incubation with 50 µl of 3, 3’, 5, 5’ tetramethylbenzidine (TMB, Sigma) for 30 min, followed by the addition of 50 µl of Stop reagent (Sigma). Absorbance at 450 nm was measured with a Synergy 4 Hybrid Multi-Mode Reader (BioTek, Winooski, VT, USA). To calculate EC_50_ values of the mAbs, absorbance at 450 nm was measured with SpectraMax Paradigm (Molecular Devices, Sunnyvale, CA, USA) and fitted to a four-parameter logistic model.

For kinetic experiments, HRP-labeled E5415A and E5415B described above were used. To determine the dissociation rate constant (*k*_-1_) of the mAbs, HRP-labeled E5415A and E5415B in 40% Block Ace were added to 96-well plates, where mAQP4-M23-expressing 4311 or M23L-mAQP4-M1-expressing 6111 cells had been seeded and fixed, and incubated at 4˚C overnight so that the binding of HRP-labeled antibody to AQP4 reached equilibrium. Dissociation of the HRP-labeled antibody was initiated either by adding 1000 times higher concentration of unlabeled antibody to the wells to prevent re-association of the HRP-labeled antibody or by removing free HRP-labeled antibody from the buffer after washing twice with PBS (dilution). Bound HRP-labeled antibodies at selected time points after initiating dissociation were measured. By plotting ln(B_t_/B_0_), where B_t_ and B_0_ are binding at time t and the binding at the start of dissociation (t = 0) at room temperature, versus the dissociation time, -*k*_-1_ values were obtained as the slope (Supplemental Fig. S1A-D). To determine the association rate constant (*k*_+1_), various concentrations of HRP-labeled mAbs in 40% Block Ace were added to 96-well plates, where mAQP4-M23-expressing 4311 had been seeded and fixed, and incubated at room temperature for various periods of time. The observed onset rate constant (*k*_obs_) values were given by plotting ln(B_eq_/(B_eq_ - B_t_)), where B_eq_ is the equilibrium binding, versus the association time as the slope (Supplemental Fig. S1E, F). *k*_+1_ (and also *k*_-1_) values were obtained by plotting *k*_obs_ versus concentration of the mAb as the slope (and the intercept, respectively). The *k*_-1_/*k*_+1_ ratio gives the equilibrium dissociation constant (*K*_D_).

*Analysis of AQP4 degradation*

To identify pathways for degradation of AQP4, 100 nM bafilomycin A1 (Merck Millipore, Billerica, MA, USA), an inhibitor for vacuolar-type H^+^-ATPase, which inhibits proteases in lysosomes due to an increase in pH value, or 1 μM MG-132 (Merck Millipore), an inhibitor for proteasomes, for CHO cells was added to culture media and incubated for 24 h. Cell lysates (30-50 µg) were subjected to Western blotting.

Confocal microscopy

For live imaging of CHO cells stably expressing AQP4, subconfluent cells seeded onto 3.5-cm glass base dishes (AGC Techno Glass Co., Ltd., Shizuoka, Japan) were incubated with Alexa-Fluor-555-labeled E5415A, or E5415B, for up to 3 h under observation with an Olympus FV1000 confocal microscope with 60×/NA1.2 objectives (Olympus, Tokyo, Japan). The cells treated with labeled antibodies for 24 h were also observed. Fluorescence of EGFP expressed in stable cells and labeled antibodies were detected on excitations at 488 nm and 543 nm, respectively, and five z-stacks spanning approximately 3 µm were captured.

*Cytotoxicity assays*

To assess complement-dependent cytotoxicity (CDC), survival rates of 6111 (M1) and 4311 (M23) cells were analyzed after exposure to mAbs E5415A and E5415B in the presence or absence of rabbit complement by means of a tetrazolium-based cell viability assay (Cell Counting Kit-8, Dojindo Molecular Technologies, Tokyo, Japan). Stable CHO-cell clones were seeded onto 96-well plates at a density of 1.2 × 10^4^ and grown at 37°C for 6–36 h. These cells were treated with each antibody (2 μg/ml) just before complement administration. Then rabbit serum (Sigma) was added to the cells. After 90-min incubation at 37°C, all of the medium in each plate was replaced with fresh growth medium containing 10% water soluble tetrazolium salts (WST)-8/1-methoxy phenazine methosulphate (PMS) solution. The cells were further incubated for 2–3 h at 37°C, followed by measurement for absorbance at 450 nm using the Synergy 4 Hybrid Multi-Mode Reader. Cell viabilities were calculated as percentages of cells grown in growth medium throughout the experiment in the absence of each antibody or complement.

*Statistical analysis*

Statistical analysis was performed using JMP ver. 11.0.0 (SAS Institute Inc., Cary, NC, USA). Data were analyzed using one-way ANOVA followed by the Tukey-Kramer method.

**Results**

*Characterization of E5415A and E5415B*

We first performed a saturation binding experiment using several CHO-cell clones expressing mAQP4 (Supplemental Fig. S1A) fixed with 4% PFA without permeabilization. Both E5415A and E5415B bound to the M23-expressing 4311 cells (Supplemental Fig. S1A, lane 1) with similar avidity (Supplemental Fig. S2A, Table 1). The expression level of mAQP4 M23 severely affected the binding capacities of these antibodies, whereas it did not impair their avidities, displaying a 2.5-3-fold leftward shift of EC_50_ values for these antibodies (Supplemental Fig. S2B and Table 1) as determined using 3113 cells, a low-expression clone expressing the mAQP4 M23 isoform (Supplemental Fig. S1A, lane 2). On the other hand, although E5415A could also bind to 6111 cells, a line expressing M1 alone (Supplemental Fig. S1A, lane 3), its avidity for mAQP4 M1 was much lower as compared with that for M23 (Supplemental Fig. S2C, Table 1). In accordance with the initial screening process, E5415B showed little binding to 6111 cells up to 10 µg/ml (Supplemental Fig. S2C). When we used cells stably transfected with cDNA encoding wild-type M1, which express not only M1 but also small amounts of M23 (Fig. 1A, lane 4, clone 1132), both E5415A and E5415B bound to the cells (Supplemental Fig. 2D). However, the maximal binding of E5415B for this clone was only 48.6% of that of E5415A. The binding avidity of E5415A for 1132 cells was intermediate between those for cells expressing M23 alone (4311 and 3113) and M1 alone (6111), while the avidity of E5415B for 1132 cells was relatively high as compared with that of E5415A (Supplemental Fig. S2D, Table 1). We also examined the binding properties of these antibodies in a stable clone expressing both M1 and M23 isoforms at a ratio of 1:1 (Supplemental Fig. S1A, lane 5), designated I911 (Supplemental Fig. S2E). As expected, both antibodies bound to these cells with avidities similar to those observed in clone 1132 (Table 1 and Supplemental Fig. S2D, E). The relatively lower capacities of these antibodies for I911 cells probably reflect the relatively low expression level of AQP4 in these cells (Supplemental Fig. S1A, lane 5). The maximal binding of E5415B for I911 cells was 51.7% of that of E5415A (Supplemental Fig. S2E), which is almost the same level as observed in 1132 cells.

Taken together, OAP formation of AQP4 was advantageous to the binding of both E5415A and E5415B to AQP4, which is also observed in the binding of the vast majority of NMO-IgGs to AQP4 [3-8]. Expression of the M1 isoform also produced another binding site for E5415A, with a relatively low avidity for this antibody (Table 1). This binding site contributed little to the binding of E5415B, indicating that the OAP formation of AQP4 is indispensable for E5415B to recognize AQP4 expressed on the surface of mammalian cells.

We further analyzed the binding properties of E5415A and E5415B by performing a kinetic experiment to find the rates of association and dissociation of the mAbs to/from AQP4. We first determined the dissociation rate constants (*k*_-1_) of the mAbs for binding to the M23 isoform expressed on fixed CHO-cell surface (4311 cells). Fixed 4311 cells on 96-well plates were incubated with 10 ng/ml of HRP-labeled E5415A or E5415B at 4˚C overnight until the binding of the HRP-labeled mAbs reached equilibrium. Then dissociations were initiated either by washing the well with PBS followed by incubation with buffer lacking the HRP-labeled antibody (dilution), or by addition of an excess (10 μg/ml) of unlabeled mAbs to the solution to avoid re-association of the HRP-labeled antibody. When the dissociation was initiated by dilution, both HRP-labeled E5415A and E5415B slowly dissociated from the cells expressing M23 alone (Supplemental Fig. S3A, B, open circles). *k*_-1_ values for HRP-labeled E5415A and E5415B were 1.75 × 10^-3^ min^-1^ and 1.99 × 10^-3^ min^-1^, respectively (Supplemental Fig. S4A, C). When the dissociation was initiated by adding an excess of unlabeled mAbs, the rate of dissociation was accelerated both in HRP-labeled E5415A and in HRP-labeled E5415B (Supplemental Fig. S3A, B; Supplemental Fig. S4B, D), indicating that the actual dissociation rate of the mAbs is relatively rapid and that the maintenance of binding of these HRP-labeled antibodies involves ‘re-association process’ of them. Importantly, the dissociation of HRP-labeled E5415A from AQP4 was similarly accelerated and completely displaced not only by an excess of unlabeled E5415A but also of unlabeled E5415B (Fig. 3A and Supplemental Fig. S1B, blue symbols) and vice versa (Fig. 3B and Supplemental Fig. S1D, red symbols), indicating that E5415A and E5415B share the binding site on cells expressing M23 isoform alone (a common binding site).

Next we determined the association rate constants (*k*_+1_) by adding various concentrations of HRP-labeled mAbs to plates, where the cells expressing M23 alone (4311 cells) had been seeded and fixed. Both HRP-labeled E5415A and E5415B were similarly bound to the cells (Supplemental Fig. S3C, D; Supplemental Fig. S4 E, F), and *k*_+1_ values for HRP-labeled E5415A and E5415B were 2.32 × 10^-5^ pM^-1^·min^-1^ and 2.89 × 10^-5^ pM^-1^·min^-1^, respectively (Supplemental Fig. S4G). Supplemental Fig. S4G also shows the *k*_-1_ values for HRP-labeled E5415A and E5415B (5.52 × 10^-3^ min^-1^ and 5.62 × 10^-3^ min^-1^, respectively), which were rather similar to those obtained by dissociation experiments by dilution (Supplemental Fig. S4A and C, respectively). The obtained *K*_D_ values for HRP-labeled E5415A and E5415B were 238 pM and 194 pM, respectively, which are roughly in accordance with the EC_50_ values obtained by saturation binding experiments (Supplemental Fig. S2A and Table 1).

It should be noted that, as shown in Supplemental Fig. S3E, dissociation of bound HRP-labeled E5415A from cells expressing the M1 isoform alone (6111 cells) was greatly enhanced and completely displaced not only by unlabeled E5415A but also by unlabeled E5415B, which did not show obvious binding to 6111 cells in the saturation binding experiment (Supplemental Fig. S2C). This suggests that E5415B can also interact with AQP4 in the absence of OAPs but that it dissociates very rapidly during the incubation with the secondary antibody followed by washing with PBS in saturation binding experiments.

These observations suggest that the binding of both E5415A and E5415B to AQP4 involves a frequent dissociation/re-association process and that OAP formation of AQP4 contributes to the enhancing ‘re-association process’ of both E5415A and E5415B to AQP4, providing them with a common binding site. Our findings also suggest that the E5415A-specific binding site demonstrated in the saturation binding experiments is also shared by E5415A and E5415B but that the dissociation of E5415B from E5415A-specific binding site is too rapid to detect the binding of E5415B to M1 by the saturation binding experiments, which include long incubation with the secondary antibody followed by extensive washing with PBS.

*E5415A enhanced endocytosis of not only M1 but also M23 in CHO cells.*

To examine the consequences of the binding of E5415A and E5415B to the ECDs of AQP4, we performed live imaging of Alexa-Fluor-555-labeled mAbs in CHO cells expressing mAQP4. Both labeled antibodies rapidly bound to the surface of 4311 cells expressing M23 alone (Supplemental Fig. S5A, D). The bindings of these labeled antibodies to the cell surface were sustained for at least 24 h. During incubation with these antibodies, intracellular accumulation of fluorescence was also observed in these cells, indicating that AQP4 underwent endocytosis (Supplemental Fig. S5A, D). Fluorescence-labeled E5415A also rapidly bound to the cells expressing M1 alone (6111 cells) and accumulated inside the cells (Supplemental Fig. S5B). After 24-h incubation, fluorescence of E5415A was mainly detected inside the cells (Supplemental Fig. S5B). Western blotting of lysate from 6111 cells expressing M1 alone demonstrated that incubation with E5415A for 24 h greatly reduced AQP4 protein in these cells (Supplemental Fig. S6B, lane 3), while C9401, a mAb specific to hAQP4 [16], did not (Supplemental Fig. S6B, lane 2). However, when 6111 cells were treated with bafilomycin A1, an inhibitor for lysosomal function, simultaneously with E5415A for 24 h, the level of AQP4 was restored (Supplemental Fig. S6B, lane 4), indicating that E5415A strongly enhances endocytosis and the subsequent lysosomal degradation of AQP4 in cells expressing M1. Although Alexa-Fluor-555-labeled E5415B was slightly detected on the surface and in the intracellular compartments of 6111 cells (Supplemental Fig. S5E, Supplemental Movie S4), E5415B did not reduce the level of AQP4 in these cells (Supplemental Fig. S6B, lane 5), which is consistent with the limited binding of E5415B to cells expressing M1 alone as examined by ELISA Supplemental Fig. S2C). Using Western blotting, we also examined the effect of these antibodies on the level of AQP4 in cells expressing M23 alone (4311 cells). In contrast to the results using 6111 cells, neither E5415A nor E5415B reduced the level of AQP4 in 4311 cells (Supplemental Fig. S6A, lanes 3, 5). Interestingly, a significant increase of AQP4 was observed in 4311 cells treated simultaneously with these antibodies and bafilomycin A1 (Supplemental Fig. S6A, lanes 4, 6). Importantly, treating cells expressing M23 alone with bafilomycin A1 was enough to increase the level of AQP4 (Supplemental Fig. S7A and B, lane 3), whereas there was no significant change in the level of AQP4 in cells expressing M1 alone treated solely with bafilomycin A1 (Supplemental Fig. S7C and D, lane 3), This indicates that when M23 is expressed alone, a significant level of AQP4 undergoes constitutive endocytosis, which is not altered by binding of antibodies. We further examined the effect of these antibodies on cells expressing both M1 and M23 (I911 cells), which mimic cells such as astrocytes endogenously expressing AQP4. Similar to the results observed with cells expressing M23 alone, both E5415A (Supplemental Fig. S5C) and E5415B (Supplemental Fig. S5F) bound to the cells, and intracellular accumulation of fluorescence was also observed. In contrast to the results using cells expressing M23 alone, 24-h treatment with labeled E5415A resulted in the localization of fluorescence, mainly in the intracellular compartment and signals on the cell surface were obscure (Supplemental Fig. S5C). On the other hand, similar to its binding to cells expressing M23 alone, labeled E5415B clearly localized on the cell surface of I911 cells even after 24 h of incubation (Supplemental Fig. S5F). Consistent with these observations, E5415A drastically reduced the level of both M1 and M23, which was restored by simultaneous treatment with bafilomycin A1 (Supplemental Fig. S6C, lanes 3, 4), while E5415B showed little effect on the level of AQP4 (Supplemental Fig. S6C, lane 5) in these cells, despite its actual binding to cell-surface AQP4. Thus, these results indicate that E5415A strongly enhances endocytosis of both M1 and M23 of mAQP4 as long as CHO cells express the M1 isoform. E5415B contributed much less to the enhancement of endocytosis of AQP4 in any cell line, suggesting that the binding of the E5415A to the E5415A-specific binding site evokes endocytosis of AQP4.

*E5415A induced complement-dependent cytotoxicity in CHO cells expressing M1 alone.*

Phuan et al. had demonstrated that a mAb rAb58, which is derived from clonally expanded plasmablasts in cerebrospinal fluid of an NMO patient, and binds to both M1 and M23 with similar affinity in a monovalent manner [5], induced no complement-dependent cytotoxicity (CDC) in cells expressing the M1 isoform alone due to lack of clustering of IgG necessary for multivalent binding of complement C1q [9]. Thus, we examined whether E5415A induces CDC in CHO cells expressing M1 alone. In the absence of a complement, neither E5415A nor E5415B showed cytotoxicity in cells expressing AQP4, regardless of its isoforms (Supplemental Fig. S 9A and B, open columns). However, they clearly induced CDC in cells expressing M23 alone (Supplemental Fig. S 9A, solid columns) because OAP formation of AQP4 is advantageous to clustering of bound IgG, which is in agreement with Phuan et al. [9]. In contrast to their results using rAb58, E5415A induced CDC in cells expressing M1 alone as well (Supplemental Fig. S 9B), indicating that the binding of E5415A to AQP4 resulted in clustering of AQP4 as well as E5415A itself on the cells. On the other hand, E5415B induced CDC in CHO cells expressing M1 alone to a much lesser extent due to its limited binding to M1 (Supplemental Fig. S 9B). Thus, these results strongly support the idea that E5415A promotes clustering of AQP4 by cross-linking more than a tetramer or an array of AQP4. In addition, these findings imply that E5415A binds to AQP4 in a bivalent manner cross-linking two AQP4 tetramers rather than doing so monovalently.

**Discussion**

Our kinetic experiments revealed that the dissociation rates of both mAbs from OAPs (cells expressing M23 alone) were much higher when an excess of unlabeled competitor coexisted with the HRP-labeled antibody than when the HRP-labeled antibody was removed from incubation buffer (Supplemental Fig. S3 and S4). This finding suggested the involvement of relatively rapid dissociation followed by re-association in their binding to AQP4. Therefore, to maintain the long half-life of their binding to AQP4 observed in dissociation by the removal of HRP-labeled antibody from incubation buffer (dilution), the re-association rates of the mAbs have to be extremely high to achieve continuous local dissociation/re-association cycles. Such cycles would prevent their diffusion into the solution despite quite a low concentration of the once-dissociated labeled antibody. To explain the mechanism of binding of these mAbs, we propose the model shown in Supplemental Fig. S11. In this model, intrinsic affinities between the epitope and the paratope of E5415A and E5415B are very low; therefore, when they bind to AQP4 in a monovalent manner, they easily dissociate from AQP4 (Supplemental Fig. S11A, left). But when AQP4 tetramers are incorporated into an array, their position and distance become quite suitable for a bivalent binding of the mAbs, enhancing the transition from monovalent to bivalent (Supplemental Fig. S11A, right), which then tightens the binding of mAbs to AQP4, cross-linking two tetramers. A similar example of binding of an antibody has been demonstrated using a mAb P20.1 recognizing the 6-residue sequence of human protease activated receptor-4 N-terminal peptide [10, 11]. The intrinsic affinity of P20.1 toward antigenic peptide was low due to its fast dissociation rate, as determined by surface plasmon resonance analysis using its Fab fragment [10]. The binding avidity of P20.1 to the antigenic peptide was augmented by concatenation of the recognition sequence; augmentation of the avidity depended on the number of repeat and the distance between the recognition motifs in the peptide [10, 11]. Thus, OAP formation of AQP4 produces the same effect, strengthening the binding between mAbs and the ECDs of AQP4 as the concatenated antigen peptide for P20.1. As observed in the kinetic experiments (Supplemental Fig. S3), in the presence of an excess of competitor, HRP-labeled mAbs rapidly dissociated from AQP4. In this situation, in addition to re-association of the free labeled mAb, transition from monovalent to bivalent binding (Supplemental Fig. S11B, purple) is also blocked by the unlabeled mAb (Supplemental Fig. S11B, brown). Then the binding of the labeled mAbs is kept in a monovalent state, which accelerates their dissociation Supplemental Fig. S11B, indicated with red arrows). In addition, according to this model, it is highly likely that the E5415A-specific binding site demonstrated in the saturation binding experiments is produced by the flexibility of E5415A in position and distance between two AQP4 tetramers to cross-link. Our functional analyses demonstrating an ability of E5415A to form large AQP4 cluster strongly support this model. Since the initial association rates of E5415A and E5415B were similar, even in the absence of OAP formation of AQP4 (Supplemental Fig. S3E), we hypothesize that E5415B cannot stably bind to the E5415A-specific site because a vast majority of the position and distance between two AQP4 tetramers outside the arrays is inappropriate for E5415B to achieve bivalent binding, causing rapid dissociation of either Fab fragment of E5415B from the epitope, or slow transition from monovalent to bivalent.

**References**

[1] K. Miyazaki, Y. Abe, H. Iwanari, Y. Suzuki, T. Kikuchi, T. Ito, J. Kato, O. Kusano-Arai, T. Takahashi, S. Nishiyama, H. Ikeshima-Kataoka, S. Tsuji, T. Arimitsu, Y. Kato, T. Sakihama, Y. Toyama, K. Fujihara, T. Hamakubo, M. Yasui, Establishment of monoclonal antibodies against the extracellular domain that block binding of NMO-IgG to AQP4, J Neuroimmunol 260 (2013) 107-116.

[2] A. Rossi, F. Pisani, G.P. Nicchia, M. Svelto, A. Frigeri, Evidences for a leaky scanning mechanism for the synthesis of the shorter M23 protein isoform of aquaporin-4: implication in orthogonal array formation and neuromyelitis optica antibody interaction, J Biol Chem 285 (2010) 4562-4569.

[3] G.P. Nicchia, M. Mastrototaro, A. Rossi, F. Pisani, C. Tortorella, M. Ruggieri, A. Lia, M. Trojano, A. Frigeri, M. Svelto, Aquaporin-4 orthogonal arrays of particles are the target for neuromyelitis optica autoantibodies, Glia 57 (2009) 1363-1373.

[4] F. Pisani, M. Mastrototaro, A. Rossi, G.P. Nicchia, C. Tortorella, M. Ruggieri, M. Trojano, A. Frigeri, M. Svelto, Identification of two major conformational aquaporin-4 epitopes for neuromyelitis optica autoantibody binding, J Biol Chem 286 (2011) 9216-9224.

[5] J.M. Crane, C. Lam, A. Rossi, T. Gupta, J.L. Bennett, A.S. Verkman, Binding affinity and specificity of neuromyelitis optica autoantibodies to aquaporin-4 M1/M23 isoforms and orthogonal arrays, J Biol Chem 286 (2011) 16516-16524.

[6] K. Miyazaki, Y. Abe, H. Iwanari, Y. Suzuki, T. Kikuchi, T. Ito, J. Kato, O. Kusano-Arai, T. Takahashi, S. Nishiyama, H. Ikeshima-Kataoka, S. Tsuji, T. Arimitsu, Y. Kato, T. Sakihama, Y. Toyama, K. Fujihara, T. Hamakubo, M. Yasui, Establishment of monoclonal antibodies against the extracellular domain that block binding of NMO-IgG to AQP4, J Neuroimmunol 260 (2013) 107-116.

[7] F. Pisani, A. Sparaneo, C. Tortorella, M. Ruggieri, M. Trojano, M.G. Mola, G.P. Nicchia, A. Frigeri, M. Svelto, Aquaporin-4 autoantibodies in Neuromyelitis Optica: AQP4 isoform-dependent sensitivity and specificity, PLoS One 8 (2013) e79185.

[8] G.P. Owens, A. Ritchie, A. Rossi, K. Schaller, S. Wemlinger, H. Schumann, A. Shearer, A.S. Verkman, J.L. Bennett, Mutagenesis of the aquaporin 4 extracellular domains defines restricted binding patterns of pathogenic neuromyelitis optica IgG, J Biol Chem 290 (2015) 12123-12134.

[9] P.W. Phuan, J. Ratelade, A. Rossi, L. Tradtrantip, A.S. Verkman, Complement-dependent cytotoxicity in neuromyelitis optica requires aquaporin-4 protein assembly in orthogonal arrays, J Biol Chem 287 (2012) 13829-13839.

[10] T. Nogi, T. Sangawa, S. Tabata, M. Nagae, K. Tamura-Kawakami, A. Beppu, M. Hattori, N. Yasui, J. Takagi, Novel affinity tag system using structurally defined antibody-tag interaction: application to single-step protein purification, Protein Sci 17 (2008) 2120-2126.

[11] S. Tabata, M. Nampo, E. Mihara, K. Tamura-Kawakami, I. Fujii, J. Takagi, A rapid screening method for cell lines producing singly-tagged recombinant proteins using the "TARGET tag" system, J Proteomics 73 (2010) 1777-1785.

**Table 1 Binding properties of mAbs against the extracellular domains of mAQP4 determined by saturation binding experiments.**

| CHO-cell clones | AQP4 isoform(s) | EC_50_ (pM) | |
| --- | --- | --- | --- |
|  |  | E5415A | E5415B |
| 4311 | M23 (high) | 72.2 ± 17.3 (n = 8) | 63.4 ± 31.2 (n = 8) |
| 3113 | M23 (low) | 24.0 ± 7.05 (n = 8) | 25.1 ± 3.61 (n = 6) |
| 6111 | M1 (high) | 693 ± 227 (n = 8) |  |
| 1132 | M1 > M23 (high) | 243 ± 58.0 (n = 6) | 152 ± 29.5 (n = 6) |
| I911 | M1 = M23 (low) | 276 ± 95.2 (n = 6) | 120 ± 29.8 (n = 6) |

Values were means ± SD of 6-8 independent experiments.
